# Lethal Mutagenesis of Hepatitis C Virus Induced by Favipiravir

**DOI:** 10.1371/journal.pone.0164691

**Published:** 2016-10-18

**Authors:** Ana I. de Ávila, Isabel Gallego, Maria Eugenia Soria, Josep Gregori, Josep Quer, Juan Ignacio Esteban, Charles M. Rice, Esteban Domingo, Celia Perales

**Affiliations:** 1 Centro de Biología Molecular “Severo Ochoa” (CSIC-UAM), Consejo Superior de Investigaciones Científicas (CSIC), Campus de Cantoblanco, 28049, Madrid, Spain; 2 Centro de Investigación Biomédica en Red de Enfermedades Hepáticas y Digestivas (CIBERehd), Barcelona, Spain; 3 Liver Unit, Internal Medicine, Laboratory of Malalties Hepàtiques, Vall d’Hebron Institut de Recerca-Hospital Universitari Vall d´Hebron, (VHIR-HUVH), Universitat Autònoma de Barcelona, 08035, Barcelona, Spain; 4 Roche Diagnostics, S.L., Sant Cugat del Vallés, Spain; 5 Universitat Autónoma de Barcelona, Barcelona, Spain; 6 Center for the Study of Hepatitis C, Laboratory of Virology and Infectious Disease, The Rockefeller University, New York, United States of America; Kaohsiung Medical University Chung Ho Memorial Hospital, TAIWAN

## Abstract

Lethal mutagenesis is an antiviral approach that consists in extinguishing a virus by an excess of mutations acquired during replication in the presence of a mutagen. Here we show that favipiravir (T-705) is a potent mutagenic agent for hepatitis C virus (HCV) during its replication in human hepatoma cells. T-705 leads to an excess of G → A and C → U transitions in the mutant spectrum of preextinction HCV populations. Infectivity decreased significantly in the presence of concentrations of T-705 which are 2- to 8-fold lower than its cytotoxic concentration 50 (CC_50_). Passaging the virus five times in the presence of 400 μM T-705 resulted in virus extinction. Since T-705 has undergone advanced clinical trials for approval for human use, the results open a new approach based on lethal mutagenesis to treat hepatitis C virus infections. If proven effective for HCV *in vivo*, this new anti-HCV agent may be useful in patient groups that fail current therapeutic regimens.

## Introduction

Lethal mutagenesis is an antiviral approach consisting of achieving viral extinction by an excess of mutations [1–6]. It is an application of the error threshold relationship of quasispecies theory that can be applied to finite populations of viruses in changing fitness landscapes [7]. We were interested in exploring lethal mutagenesis for the treatment of HCV infections, based on the evidence that ribavirin (1-*β*-D-ribofuranosyl-1-*H*-1,2,4-triazole-3-carboxamide), an important component of several anti-HCV therapies, might be exerting its antiviral action partly through lethal mutagenesis [8,9]. Effective antiviral lethal mutagenesis therapy will require additional agents that mutagenize the virus and not the cells, and provide an advantage over standard non-mutagenic inhibitors and their combinations.

Favipiravir (T-705; 6-fluoro-3-hydroxy-2-pirazinecarboxamide) is one of several pyrazinecarboxamide derivatives that display a broad spectrum antiviral activity against RNA viruses. Work by Furuta and colleagues has documented that T-705 is active against influenza virus, and with lower potency also against poliovirus, rhinovirus and respiratory syncytial virus [10,11], and that T-1105 (3-hydroxy-2-pyrazinecarboxamide) inhibited foot-and-mouth disease virus (FMDV) replication in cell culture and *in vivo* [12]. T-1106, the nucleoside derivative of T-1105, inhibited replication of bovine viral diarrhea virus and HCV [13]. RNA viruses as diverse as picornaviruses, alphaviruses, flaviviruses, rhabdoviruses, orthomyxoviruses, paramyxoviruses, arenaviruses, hantaviruses and bunyaviruses are inhibited by members of this family of antiviral agents [14–28]. Moreover, T-705 potentiated the anti-influenza activity of oseltamivir [24] and the anti-arenavirus activity of ribavirin [29,30].

Present evidence suggests that these inhibitors target the viral RNA-dependent RNA polymerase (RdRp) resulting in inhibition of viral RNA synthesis [31,32]. T-705 is converted into nucleotide derivatives inside the cell, and T-705-4-ribofuranosyl-5’-triphosphate (T-705-RTP) inhibited the influenza virus polymerase in a GTP-competitive manner [11]. In replicating influenza RNA, T-705-RTP can be ambiguously recognised as G or A, and the consecutive incorporation of two T-705-RMP residues in the RNA produced chain termination [33].

The ambiguous base pairing of T-705-RTP is consistent with a dominance of G → A and C → U transitions in viral RNA that led to lethal mutagenesis of influenza virus [34]. T-705 induced also lethal mutagenesis of norovirus in cell culture and *in vivo*, although in this case progeny RNA acquired an excess of A → G and U → C transitions [35]. In the present study we show that T-705 is a mutagenic agent for HCV that produces an excess of G → A and C → U transitions, leading to loss of infectivity through a decrease of specific infectivity. The results reinforce the possibility of lethal mutagenesis as an alternative antiviral design to treat HCV infections.

## Materials and Methods

### Cells and viruses

The origin of Huh-7.5, Huh-7-Lunet, Huh-7.5 reporter cell lines and procedures for cell growth in Dulbecco’s modification of Eagle’s medium (DMEM), have been previously described [36,37]. Infected and uninfected cells were cultured at 37°C and 5% CO_2_. The viruses used in the experiments reported here are HCVcc [Jc1FLAG2(p7-nsGluc2A)] (a chimera of J6 and JFH-1 from genotype 2a) and GNN [GNNFLAG2(p7-nsGluc2A)] (carrying a mutation in the NS5B RNA-dependent RNA polymerase rendering it replication-defective) [38]. To control for the absence of contamination, the supernatants of mock-infected cells, which were maintained in parallel with the infected cultures, were titrated; no infectivity in the mock-infected cultures was detected in any of the experiments.

### Production of viral progeny and titration of infectivity

The procedures used to prepare the initial virus stock HCV p0 and for serial infections of the human hepatoma Huh-7.5 cells have been previously described [39]. Briefly, Huh-7-Lunet cells were electroporated with 10 μg of the infectious transcript of HCVcc (Jc1 or the negative control GNN) (Gene Pulser Xcell electroporation system; Bio-Rad; 260 volts, 950 μF). Electroporated cells were then passaged every 3–4 days without allowing the cells to reach confluence; passages were continued until 30 days post-electroporation, and the cell culture supernatants were pooled. The virus was then concentrated 20 times using 10,000 MWCO spin columns (Millipore) as instructed by the manufacturer, and stored in aliquots (at -70°C). To increase virus infectivity, Huh-7.5 reporter cells were infected with concentrated virus stocks at a MOI of 0.5 TCID_50_/cell, and the cells were passaged to obtain the working viral stock HCV p0. The infection of Huh-7.5 cells with HCV p0 can be sustained for at least 100 serial passages [39]. For titration of HCV infectivity, serially diluted cell culture supernatants were applied to Huh-7.5 cells and 3 days post-infection the cells were washed with PBS, fixed with ice-cold methanol, and stained using anti-NS5A monoclonal antibody 9E10, as previously described [39,40].

### Treatment with favipiravir (T-705)

A solution of T-705 (Atomax Chemicals Co. Ltd) was prepared at a concentration of 20 mM in H_2_O. It was sterilized by filtration, and stored at –70°C. Prior to use, the stock solution was diluted in DMEM to reach the desired concentration. Huh-7.5 reporter cells were pretreated with the appropriate drug concentrations (or with DMEM without drug) during 16 h prior to infection. Then, 4 x 10^5^ Huh-7.5 reporter cells were infected (or mock infected) with 1.2 x 10^4^ TCID_50_ of HCV p0; the adsorption time was 5 h, and the infection continued for 72 to 96 h in the absence or presence of T-705. For successive viral passages, 4 x 10^5^ Huh-7.5 reporter cells were infected with 0.5 ml of the supernatant from the previous infection; the MOI ranged between 0.6 and 5 x 10^−5^ TCID_50_/cell; each MOI can be calculated from the infectivity values given for each experiment.

### Toxicity assays

The CC_50_ of T-705 was measured by seeding 96-well plates with Huh-7.5 cells to 70% confluence and exposing the cells to a range of T-705 concentration for up to 142 h. MTT [3-(4,5-dimethylthiazol-2-yl)-2,5-diphenyltetrazolium bromide] was added to each well at a final concentration of 500 μg/ml; 4 h later crystals were dissolved in 100 μl of DMSO and the O.D. measured at 550 nm; 50% cytotoxicity was calculated from four different determinations as previously described [38].

### Inhibitory concentration

The IC_50_ of T-705 was calculated relative to the progeny infectivity of the untreated controls (defined as 100% infectivity), as described previously [41,42]; determinations were carried out in triplicate.

### RNA extraction, cDNA synthesis, and PCR amplification for Sanger nucleotide sequencing

Intracellular viral RNA was extracted from infected cells using the Qiagen RNeasy kit according to the manufacturer’s instructions (Qiagen, Valencia, CA, USA). RT-PCR amplification was carried out using AccuScript (Agilent), as specified by the manufacturers. NS5B genomic region was amplified using the specific oligonucleotides Jc1-NS5B-F1 (5’-TGGTCTACTTGCTCCGAGGAGGAC-3’) and Jc1-NS5B-R4 (5’-AGTTAGCTATGGAGTGTACCTAG-3’). Nucleotide sequences of genomic HCV RNA were determined using the 23 ABI 3730XLS sequencer. To evaluate the complexity of mutant spectra, HCV RNA was extracted as described above and subjected to RT-PCR to amplify the NS5B-coding region as previously described [39]. Amplification products were analyzed by agarose gel electrophoresis using HindIII-digested Ф-29 DNA as molar mass standard. Negative controls (amplifications in the absence of RNA) were included in parallel to ascertain the absence of contamination by template nucleic acids. To ensure an excess of template in the RT-PCR amplifications for quasispecies analysis, and to avoid complexity biases due to redundant amplifications of the same initial RNA templates, amplifications were carried out with template preparations diluted 1:10, 1:100 and 1:1000; only when at least the 1:100 diluted template produced a visible DNA band was molecular cloning pursued using the DNA amplified from undiluted template [43]. Controls to ascertain that mutation frequencies were not affected by the basal error rate during amplification have been previously described [44].

### Ultra deep sequencing

For the ultra deep sequencing (UDPS) analysis (GS-Junior platform, 454 Life Sciences-Roche), reverse transcription (RT) was performed for 60 min at 40°C using Accuscript High Fidelity Reverse Transcriptase (Agilent) with a specific oligonucleotide covering the NS5A region. The products were then subjected to a PCR using Pfu Ultra II Fusion HS DNA polymerase (Agilent); the primers were composed of a specific sequence and a universal M13 primer, either upstream or downstream of the specific sequence (S1 Table). For the PCR, 5 μl of reverse transcription product were mixed with 5 μl of 10X buffer, 0.8 mM of dNTPs, 2 ng/μl of each sense and antisense primer. The initial denaturing step was at 95°C for 1 min, and it was followed by 40 cycles of a denaturing step at 95°C for 20 seconds, annealing at 60°C for 20 seconds, extension at 72°C for 1 min, and then a final extension at 72°C for 5 min.

The PCR products were then subjected to a nested PCR using Pfu Ultra II Fusion HS DNA polymerase (Agilent). The primers were composed of a complementary universal M13 primer, upstream or downstream followed by a Roche’s Validated Multiplex Identifier (MID) with oligonucleotide A or B (supplier nomenclature) at the 5’ or 3’ end of the upstream or downstream primer, respectively. For the PCR, 5 μl DNA of the previous PCR amplification mixture was added to 5 μl of a mixture containing 0.8 mM of dNTPs, 0.4 μM of sense and antisense PCR primers. The initial denaturing step was at 95°C for 1 min, and it was followed by 15 cycles of a denaturing step at 95°C for 20 seconds, annealing at 60°C for 30 seconds, extension at 72°C for 1 min, and then a final extension at 72°C for 5 min. The PCR products were purified (QIAquick Gel Extraction Kit), quantified (Pico Green Assay), and analyzed for quality (Bioanalyzer) prior to the UDPS procedure. Negative controls (without template RNA) were run in parallel to ascertain absence of contamination with undesired templates.

### Data treatment methods in ultra deep sequencing

The fasta file obtained from the 454/GS-Junior system was subjected to demultiplexing and quality filtering as previously described [45,46]. The haplotypes common to the forward and reverse strand with abundances 0.1% or higher in each strand were considered established haplotypes. The post-filter coverage of each amplicon, ranged from 4566 to 9807 reads, median 7874 and standard deviation 2075. To balance biases, the amplicons were down sampled (DS) to a common size of 4500 reads (coverage of the smallest sample), and the resulting frequencies were subjected to fringe trimming (FT), excluding haplotypes with estimated frequencies below 0.2% with 95% confidence; this procedure yielded the DSFT haplotypes [47,48].

Diversity indices were computed using the DSFT haplotypes. A set of incidence-based indices (number of haplotypes, number of mutations, and number of polymorphic sites), abundance-based indices (Shannon entropy, Gini-Simpson index, and Hill numbers of order 1,2 and infinity), functional incidence-based indices (Mfe, FAD and ^π_e_), and functional abundance-based indices (Mf minimum, Mfm and ^π) were calculated for each amplicon as previously described [47](S1 Fig). Standard deviations and confidence intervals were computed by a semiparametric bootstrap, where the haplotype frequencies are the parameters of a multinomial distribution. Each multinomial resample (2000 cycles of bootstrap) was then subjected to DSFT and the resulting haplotypes and frequencies were used to calculate diversity indices. The standard deviations were calculated as the standard deviation of the bootstrap values obtained for each index, and an approximate 95% confidence interval (CI) was computed as the basic bootstrap CI [49,50]. P-values were computed as the number of bootstrap value differences larger or equal than the observed difference; for this purpose, 10,000 bootstrap cycles were performed. The observed diversity differences were calculated with the full set of diversity indices using the DSFT haplotypes of the two amplicons to be compared. The null hypothesis is that favipiravir had no effect, that is all reads might belong to the same quasispecies; the alternative hypothesis is that favipiravir is mutagenic and increases quasispecies complexity. The null distribution is the pool of all haplotypes, prior to the DSFT procedure, with corresponding frequencies for sequences obtained in absence and presence of favipiravir. As most of the observed values of diversity lie far beyond the null distribution (see S2 Fig with boxplots), the bootstrap p-values are a conservative upper bound. An alternative approach was to consider the asymptotic normality of the difference of diversity values obtained in the bootstrap (S2 Fig), and to calculate a p-value from a normal distribution with mean and standard deviation as estimated by the bootstrap itself. Both sets of p-values were multitest-adjusted by the Bonferroni correction [51] to take into account that the full set of diversity indices was simultaneously tested. The new sequences derived from this study can be found as S3 Fig.

### Quantification of HCV RNA

Real time quantitative RT-PCR was carried out using the Light Cycler RNA Master SYBR Green I kit (Roche), according to the manufacturer’s instructions, as previously described [52]. The 5’-UTR non-coding region of the HCV genome was amplified using as primers oligonucleotide HCV-5UTR-F2 (5’- TGAGGAACTACTGTCTTCACGCAGAAAG; sense orientation; the 5’ nucleotide corresponds to genomic residue 47), and oligonucleotide HCV-5UTR-R2 (5’- TGCTCATGGTGCACGGTCTACGAG; antisense orientation; the 5’ nucleotide corresponds to genomic residue 347). Quantification was relative to a standard curve obtained with known amounts of HCV RNA, synthesized by *in vitro* transcription of plasmid GNN DNA. The specificity of the reaction was monitored by determining the denaturation curve of the amplified DNAs. Negative controls (without template RNA and RNA from mock-infected cells) were run in parallel with each amplification reaction, to ascertain absence of contamination with undesired templates.

## Results

### Inhibition of hepatitis C virus replication in hepatoma cells by T-705

**Fig 1:**
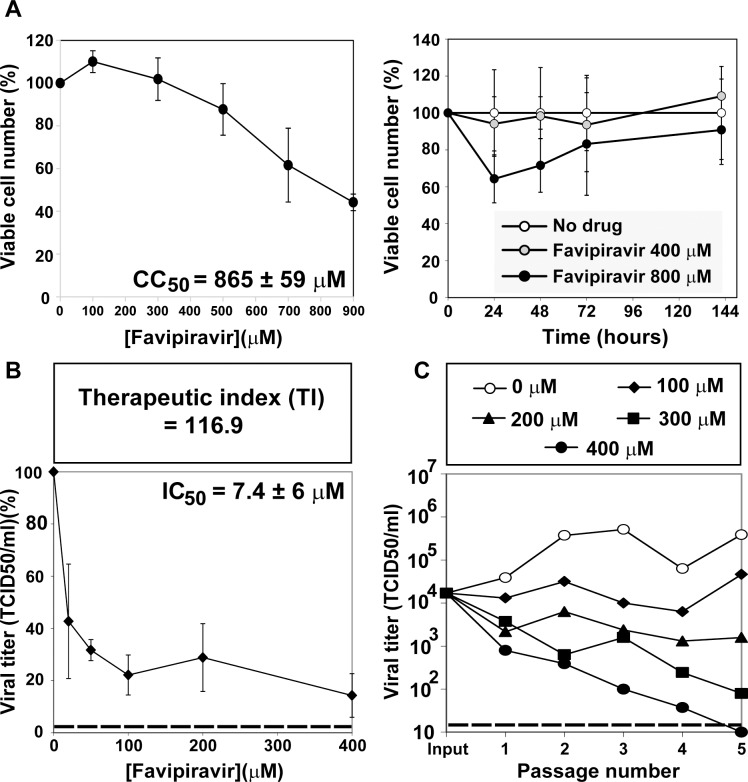
Cytotoxicity for Huh-7.5 cells, and inhibition of HCV progeny production by T-705. (A) Determinations of cytotoxic concentration 50 (CC_50_) and the effect of 400 μM and 800 μM T-705 on cell viability, (B) drug concentration required for 50% inhibition, or inhibitory concentration 50 (IC_50_); experiments were carried out in triplicate. Values and standard deviations were calculated using the program Sigma Plot. (C) Huh-7.5 reporter cells were infected with HCV p0 at a MOI of 0.03 TCID_50_/cell (4 x 10^5^ Huh-7.5 cells infected with 1.2 x 10^4^ TCID_50_), in the absence or presence of the T-705 concentrations indicated in the box. Infections with HCV GNN were carried out in parallel (negative control). Experimental conditions for cell growth, HCV infection, determination of cell viability, HCV infectivity, and serial virus passages are described in Materials and Methods. Discontinuous horizontal lines indicate the limit of detection.

The cytotoxicity of T-705 for human hepatoma Huh-7.5 cells was quantified in experiments of exposure of different drug concentrations to the cells for a fixed time, or two drug concentrations for variable times, up to 142 h. The T-705 concentration that reduced cell viability by 50% (CC_50_) was 865 ± 59 μM (Fig 1A), and the T-705 concentration that produced a 50% decrease in infectious progeny production (IC_50_) of HCV p0 was IC_50_ = 7.4 ± 6 μM (Fig 1B). These values yield a therapeutic index (TI = CC_50_ / IC_50_) of 116.9. The inhibition was sustained over at least five serial passages of the virus, in a dose-dependent manner (Fig 1C). The differences in progeny production in the absence and presence of T-705 at 200 μM, 300 μM and 400 μM concentration were statistically significant over the five passages (p = 0.007 for 200 μM, p = 0.0004 for 300 μM and p <0.0001 for 400 μM; ANOVA test). No infectivity was rescued when subjecting the cell culture supernatant from passage five in the presence of 400 μM T-705 to three blind passages in the absence of drug. Thus, T-705 is a potent inhibitor of HCV during replication in Huh-7.5 cells that can lead to virus extinction.

### Mutagenic activity of T-705 for hepatitis C virus

To investigate if the inhibition of HCV replication might be associated with a mutagenic activity for HCV, the mutant spectra of the virus passaged three times in the absence or presence of T-705 was analyzed, and several diversity indices were calculated [47]. Three amplicons of NS5A were analyzed by ultra-deep pyrosequencing (Table 1 and S1 and S2 Figs). All indices, except those denoted as being at entity level, increased significantly (p<0.01; bootstrap) when T-705 was present during replication, suggesting a mutagenic activity of this compound on HCV. Variation of indices at the entity level (Mfe and ^˄^π_e_) would require an increase in the number of mutations per haplotype induced by T-705. Despite T-705 increasing the number of genomes with any number of mutations, an increase in the number of mutations per haplotype was not observed.

**Table 1:** Ultra deep pyrosequencing analysis of HCV p0 subjected to three passages in the absence or presence of 400 μM favipiravir^a^.

		NS5A amplicon^b^		
Parameter or diversity index^c^	Favipiravir	A1 (6152–6454)	A2 (6446–6767)	A4 (6910–7252)
**Number of nucleotides sequenced**	-	2,366,733	2,589,202	1,477,987
	+	2,156,148	2,895,746	1,459,465
**Number of haplotypes**^**d**^	-	5 (2/2/0/0)	9 (7/0/1/0)	27 (23/3/0/0)
	+	30 (26/3/0/0)	33 (30/2/0/0)	66 (55/8/1/1)
**Number of different mutations**	-	6	10	24
	+	30	34	64
**Number of total mutations**	-	679	1,318	1,023
	+	1,788	2,590	2,234
**Number of polymorphic sites**	-	6	9	24
	+	29	33	64
**Dominant haplotype abundance (%)**	-	92.36	84.90	78.32
	+	76.01	72.18	55.14
^**˄**^**H**_**S**_	-	0.3374	0.6640	1.1820
	+	1.2367	1.3756	2.5032
^**˄**^**H**_**GS**_**, sample-based Gini-Simpson index**	-	0.1438	0.2731	0.3836
	+	0.4154	0.4682	0.6918
^**1**^**D (p), Hill numbers**	-	1.40	1.94	3.26
	+	3.44	3.96	12.22
^**2**^**D (p), Hill numbers**	-	1.17	1.38	1.62
	+	1.71	1.88	3.24
^**∞**^**D (p), Hill numbers**	-	1.08	1.18	1.28
	+	1.32	1.38	1.81
**Mfe, mutation frequency, entity level**	-	4.0 x 10^−3^	3.4 x 10^−3^	3.1 x 10^−3^
	+	3.5 x 10^−3^	3.2 x 10^−3^	3.4 x 10^−3^
**FAD, Functional Attribute Diversity**	-	0.16	0.49	4.33
	+	6.09	6.75	29.25
^**˄**^**π**_**e,**_ **sample nucleotide diversity, entity level**	-	7.9 x 10^−3^	6.8 x 10^−3^	6.2 x 10^−3^
	+	7.0 x 10^−3^	6.4 x 10^−3^	6.8 x 10^−3^
**Mf min, minimum mutation frequency**	-	2.5 x 10^−6^	3.9 x 10^−6^	1.6 x 10^−5^
	+	1.4 x 10^−5^	1.2 x 10^−5^	4.4 x 10^−5^
**Mf max (Mfm), maximum mutation frequency**	-	2.9 x 10^−4^	5.1 x 10^−4^	6.9 x 10^−4^
	+	8.3 x 10^−4^	8.9 x 10^−4^	1.5 x 10^−3^
^**˄**^**π, sample nucleotide diversity**	-	5.5 x 10^−4^	9.8 x 10^−4^	1.3 x 10^−3^
	+	1.6 x 10^−3^	1.7 x 10^−3^	3.0 x 10^−3^

^a^The populations analyzed correspond to passage 3 of the infections described in Fig 1.

^b^The HCV genome residue numbering corresponds to the JFH-1 genome (accession number #AB047639). The number of reads on which the parameters were calculated was 4,500 for each amplicon. Procedures are described in Materials and Methods. Mutation types are summarized in Fig 3 and their position in the HCV genome and deduced amino acid substitutions are given in S2 and S3 Tables.

^c^Diversity indices are defined and calculated as described in [47].

^d^In parenthesis the number of haplotypes with one, two, three, and four mutations is given; no haplotypes with a higher number of mutations were found.

To obtain an independent confirmation of the mutagenic activity of T-705 on HCV, the mutant spectrum of the polymerase NS5B-coding region of the same populations was analyzed by molecular cloning and Sanger sequencing. The results (Table 2) indicate a mutagenic activity of T-705, with significant increases in mutation frequencies (p<0.0001;χ^2^ test). Thus, T-705 is mutagenic for HCV. No infectivity was detected in the cell culture supernatant of HCV that was passaged five times in the presence of 400 μM T-705.

**Table 2:** Quasispecies analysis of the NS5B-coding region of hepatitis C virus population HCV p3 in the absence and presence of favipiravir^a^.

Parameter or diversity index^b^	Favipiravir	NS5B
**Number of nucleotides sequenced**	-	31,968
	+	35,520
**Number of haplotypes**^**c**^	-	9 (2/3/3/0/0/0/0/0/0)
	+	20 (1/0/6/5/3/1/3/0/1)
**Number of different mutations**	-	17
	+	69
**Number of total mutations**	-	17
	+	71
**Number of polymorphic sites**	-	17
	+	69
^**˄**^**H**_**S**_	-	1.8334
	+	2.9957
^**˄**^**H**_**GS**_**, sample-based Gini-Simpson index**	-	0.7059
	+	1.0000
^**1**^**D (p), Hill numbers**	-	6.25
	+	20
^**2**^**D (p), Hill numbers**	-	3.0
	+	20
^**∞**^**D (p), Hill numbers**	-	1.8
	+	20
**Mfe, mutation frequency, entity level**	-	1.0 x 10^−3^
	+	2.5 x 10^−3^
**FAD, Functional Attribute Diversity**	-	0.15
	+	1.51
^**˄**^**π**_**e,**_ **sample nucleotide diversity, entity level**	-	2.1 x 10^−3^
	+	4.0 x 10^−3^
**Mf min, minimum mutation frequency**	-	5.3 x 10^−4^
	+	2.0 x 10^−3^
**Mf max (Mfm), maximum mutation frequency**	-	5.3 x 10^−4^
	+	1.9 x 10^−3^
^**˄**^**π, sample nucleotide diversity**	-	1.1 x 10^−3^
	+	4.0 x 10^−3^

^a^The populations analyzed correspond to passage 3 of the infections described in Fig 1. The NS5B residues analyzed are 7667–9442. The HCV genome residue numbering corresponds to the JFH-1 genome (accession number #AB047639). Mutation types are summarized in Fig 3 and their position in the HCV genome and deduced amino acid substitutions are given in S3 Table.

^b^Diversity indices are defined and calculated as described in [47].

^c^In parenthesis the number of haplotypes with one, two, three, four, five, six, seven, eight and nine mutations is given; no haplotypes with a higher number of mutations were found.

**Fig 2:**
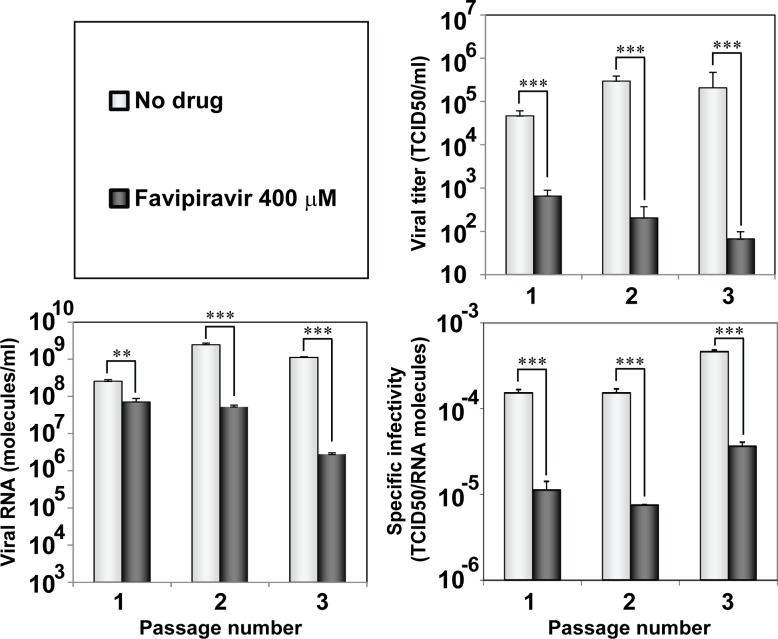
Effect of T-705 on the specific infectivity of HCV. Huh-7.5 reporter cells were infected with HCV p0 at an initial MOI of 0.03 TCID_50_/cell, in the absence or presence of 400 μM T-705; infection with GNN was performed as negative control. The infectivity values (upper right panel) have been redrawn from those shown in Fig 1(C). Extracellular viral RNA was measured by quantitative RT-PCR (bottom letf panel). Specific infectivities (bottom right panel) were calculated by dividing the infectivity by the amount of viral RNA. Statistically significant differences are indicated by three asterisks [(p<0.001); one way analysis of variance]. The range of specific infectivities determined at passages 1, 2 and 3 was 1.3 x 10^−4^ to 2.2 x 10^−5^ TCID_50_/RNA molecules for T-705 100 μM, 1.1 x 10^−5^ to 2.7 x 10^−5^ for T-705 200 μM, and 8.0 x 10^−5^ to 3.1 x 10^−6^ for T-705 300 μM. Procedures are described in Materials and Methods.

To confirm that loss of infectivity of HCV by T-705 followed a hallmark of lethal mutagenesis, the specific infectivity (the ratio between viral infectivity and the amount of genomic viral RNA) of the virus replicating at a concentration of 400 μM T-705 was calculated (Fig 2). A 13-fold to 20-fold decrease of specific infectivity occurred over the first three passages of treatment with the drug (that are those in which measurement of infectivity and viral RNA in samples of cell culture supernatant were reliable); differences were statistically significant between values in the absence and presence of the drug (p<0.0001 for passages 1 and 3, and p = 0.0001 for passage 2; t-test). In addition, treatment with T-705 did not alter the consensus genomic nucleotide sequence, again an observation made during lethal mutagenesis of viruses [53,54].

### Mutational bias evoked by T-705

**Fig 3:**
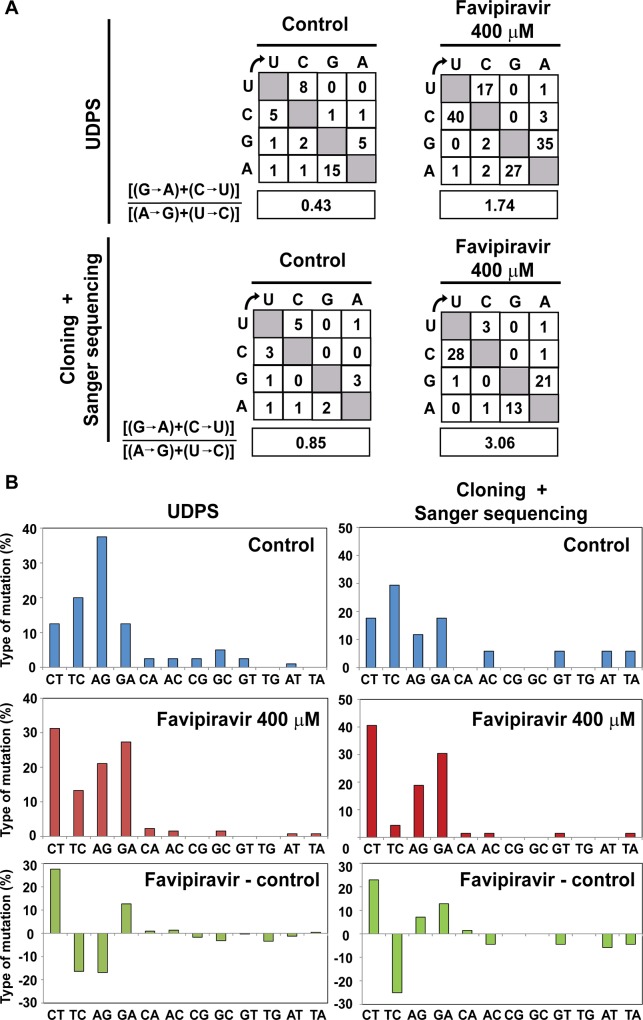
Mutational spectrum induced by favipiravir on hepatitis C virus. (A) Matrix of mutation types found in the NS5A-coding region of HCV p0 passaged three times in absence or presence of favipiravir (400 μM), based on haplotypes of three amplicons determined by UDPS, as detailed in Table 1. Below, matrix of mutation types found in the NS5B-coding region of the same viral populations, based on molecular cloning and Sanger sequencing, as detailed in Table 2. The box below each matrix quantifies the mutational bias, according to the transition type ratio shown on the left. (B) Percentage of mutation types considering 100% as the sum of all mutation types in the same populations and genomic regions analyzed in (A). The bottom panels indicate the difference in mutation types between the population passaged in presence and absence (control) of favipiravir. Procedures are detailed in Materials and Methods.

The types of the different mutations at the NS5A and NS5B regions analyzed in the populations passaged in the absence and presence of T-705 (S2, S3 and S4 Tables) indicate a predominance of C→ U and G → A transitions, with a 3.6—to 4.0- fold increase in the ratio [(G → A) + (C→ U)] / [(A → G) + (U→ C)] ratio, associated with replication in the presence of T-705 (Fig 3). Thus, T-705 is a potent mutagenic agent for HCV that produces a bias in favor of G → A and C→ U transitions preceding loss of infectivity.

## Discussion

In the present report we have shown that favipiravir (T-705) is a potent inhibitor of HCV replication in Huh-7.5 cells, with a therapeutic index (TI) value of 116.9 which is seven to nine times the value obtained previously for ribavirin in two independent determinations in the same virus-host system (TI = 12.8 [42]; TI = 15.6 [55]). According to the IC_50_ values, the inhibitory activity of T-705 for HCV is comparable to the activity exhibited against other RNA viruses [14,16,31,34,56]. The TI values for different, non-mutagenic anti-HCV agents using the same HCV p0 and Huh-7.5 cell culture system vary by orders of magnitude: 252.9, 602.4, >2000, 1.49x10^6^ and >2x10^8^ for telaprevir, cyclosporine A, sofosbuvir, daclatasvir and IFN-α, respectively [41,55,57]. Therapeutic efficacy may be different *in vivo* than in cell culture. Despite differences of values measured with HCV p0 in Huh-7.5 cells, each of the inhibitors tested has had a significant role in anti-HCV therapy.

The toxicity of T-705 for Huh-7.5 cells and the calculated CC_50_ value (Fig 1A) exclude that virus extinction (Fig 1C) was due to toxicity of T-705 for Huh-7.5 cells. The evidence that T-705 can act as a lethal mutagen includes also an increase in mutation frequency associated with a bias in favor of G→A and C→U transitions, a decrease of specific infectivity, and invariance of the consensus sequence. These are features typical of lethal mutagenesis, as previously established with several viruses and mutagenic nucleotide analogues (reviewed in [58,59]). The mutational bias evoked by T-705 is similar to that induced by ribavirin on HCV [42] and on FMDV [43,60,61]. The movement of viral genomic sequences towards extreme regions of sequence space is a critical deleterious event preceding extinction [60,62]. Our previous studies with FMDV have shown that a class of ribavirin- or 5-fluorouracil-resistant mutants harboring amino acid replacements in the viral polymerase or in non-structural protein 2C has as its mechanism of action to counteract the mutational bias induced by the mutagen [63,64]. The present study adds the important human pathogen HCV to a growing list of viral pathogens reported to be mutagenized by T-705 [34,35]. It is not clear whether the inhibition of HCV p0 replication by T-705 is exclusively a consequence of its mutagenic activity or T-705 has an inhibitory activity independent of its mutagenic activity, as previously documented for 5-fluorouracil acting on FMDV [65].

Despite the success of direct acting antiviral agents (DAAs) that can reach sustained response levels exceeding 90% [66–82], we have identified five reasons that justify exploration of new antiviral compounds to treat HCV infections: (i) There are patients who do not eliminate the virus with the new DAAs, in particular those infected with the so called “hard to treat” HCV genotypes such as genotype 3 HCV [83–85]. (ii) Inhibitor-escape mutants have been described for virtually every anti-HCV agent used alone or in combination, and their frequency is expected to increase with the extended use of new treatments, as judged by the pattern observed with HIV-1 during the AIDS pandemic. Selection of resistant mutants within individual patients or their increase during the epidemiological spread of the virus will require drugs with new mechanisms of action (reviewed in [58,59]). (iii) There are reports of patients who fail therapy and that in the breakthrough virus no resistance mutations to the drugs used in the treatment are detected [86–89]. One possibility to explain these clinical observations is that high fitness or a fitness-associated trait confers resistance to several anti-HCV agents [55,57]; high fitness viruses may be more sensitive to lethal mutagenesis than to standard inhibitors, a possibility that we are currently investigating. (iv) A recent report indicates that DAA-based treatments may induce tumor recurrence in about 27% of HCV-infected patients previously treated successfully of HCV-associated liver cancer [90]. If extended to other patient cohorts, the possibility of cancer recurrence may impose a limitation for the use of some DAAs. Although the recurrence mechanism is not known, tumor recurrence was not reported during the years in which patients were treated with pegylated interferon-alpha and ribavirin (pegIFN-α+Rib), the standard of care one decade ago. Although ribavirin has several mechanisms of activity [91–98], genetic and clinical evidences suggest that mutagenesis may be part ot its detrimental activity for HCV *in vivo* [8,9]. The possibility that lethal mutagens may extinguish HCV without the side effect of tumor recurrence is worth exploring. (v) The benefits of a treatment option depend on the HCV genotype. In the present DAA era, genotype 3 is a “hard to treat” HCV while sustained response rates of 65% to 80% were achieved after 24-week treatment with pegIFN-α+Rib (comparative efficacies for different HCV genotypes with various treatments described in [99–102], among other examples). The quasispecies dynamics of HCV [59,103–105] helps interpreting not only the existence of genotypes but also their origin and complexity. Genotypes are sets of related genomes that accumulate at some regions of sequence space due to a combination of adequate replicative and epidemiological fitness [58]. Given that antiviral efficacy is multifactorial −involving host and viral traits− it is expected that different treatments will not exhibit the same efficacy across genotypes. According to our model studies in cell culture, replicative fitness –one of the factors likely involved in genotype differentiation− is also a determinant of inhibitor efficacy [55,57]. Therefore, the available evidence suggests that if T-705 or other viral mutagens were licensed for a clinical application, it would not be possible to predict their efficacy *in vivo*, or their relative efficacy against the different existing HCV genotypes, as well as new genotypes likely to come. Assuming, however, that T-705 and ribavirin have a similar anti-HCV activity in the clinic, it is likely that the efficacy of T-705 would require its use in combination with other antiviral agents.

An advantage of considering T-705 as a potential anti-HCV inhibitor is that the drug has already undergone advanced clinical trials of efficacy and safety for treatment of other human viral diseases such as uncomplicated influenza in adults (US National Institutes of Health, identifier NCT02008344) and Ebola infection (JIKI trial, US National Institutes of Health, identifier NCT02662855 [106]). Thus, T-705 use for HCV treatment would be an example of drug repurposing, increasingly practiced in pharmacology to accelate testing and approval of drugs for new indications.

In summary, given the clinical evidence of still incomplete efficacy of the DAA-based treatments, of DAA-promoted hepatocarcinoma recurrence in patients previously subjected to successful tumor resection and treatment, and the continuing HCV diversification that will necessitate new treatments for optimal efficacy, favipiravir and other lethal mutagens may find a new role in anti-HCV treatment.

## Supporting Information

S1 Fig Barplots with diversity values for each of the three amplicons (A1, A2 and A4) and the two conditions (favipiravir, FVP and control, Ctrl). The diversity indices are abbreviated in ordinate (Hpl, number of haplotypes; nMuts, number of different mutations; PolySites, number of polymorfic sites; Mpct; dominant haplotype abundance; Shannon, ^H_S_; GiniS, ^H_GS_, sample-based Gini-Simpson index; D1, D2, Dinf, Hill numbers; Mfe, mutation frequency, entity level; FAD, Functional Attribute Diversity; Pi.e, sample nucleotide diversity, entity level; Mf min, minimum mutation frequency; Mf.max, maximum mutation frequency; Pi, sample nucleotide diversity, and their calculation is described in reference [47] of the main text. Standard deviation interval (left column), and basic bootstrap with 95% confidence intervals (CI)(right column) are shown for each index.
(PDF)

S2 Fig Histogram of null distribution bootstraped diversity differences between the populations passaged in the presence (FVP) and in the absence of favipiravir (Ctrl) with superimposed mean (dash-dot line) and normal distribution with bootstrap mean and standard deviation. The diversity index is given in the abscissa, with the same abbreviations used in S1 Fig. Density means the probability density of the corresponding distribution. The panels on the right indicate the boxplot of null distribution bootstraped diversity differences, with observed difference as red dot and red dash-dot line. The distance from this line to the boxplot, in terms of boxplot width, is an illustration of the low p-values obtained. A1, A2 and A4 mean amplicons 1, 2 and 4, respectively.
(PDF)

S3 Fig Raw data obtained from ultra-deep pyrosequencing experiments. (ZIP)

S1 Table Oligonucleotides used for the ultra deep pyrosequencing analysis of HCV p0 subjected to three passages in the absence or presence of 400 μM favipiravir. (PDF)

S2 Table Mutations, corresponding amino acid substitutions and point accepted mutation (PAM) of the NS5A-coding region in the mutant spectra HCV p0 subjected to three passages in the absence of drug analyzed by ultra deep pyrosequencing. (PDF)

S3 Table Mutations, corresponding amino acid substitutions and point accepted mutation (PAM) of the NS5A-coding region in the mutant spectra HCV p0 subjected to three passages in the presence of favipiravir (T-705) 400 μM analyzed by ultra deep pyrosequencing. (PDF)

S4 Table Mutations, corresponding amino acid and point accepted mutation (PAM) of the NS5B-coding region in the mutant spectra HCV p0 subjected to three passages in the absence or presence of 400 μM Favipiravir (T-705). (PDF)
