# Supplementary figures and images for: Lethal Mutagenesis of Hepatitis C Virus Induced by Favipiravir
Source: PLoS One. 2016 Oct 18;11(10):e0164691. doi: 10.1371/journal.pone.0164691 (PMC5068784; doi:10.1371/journal.pone.0164691)

S1 Fig

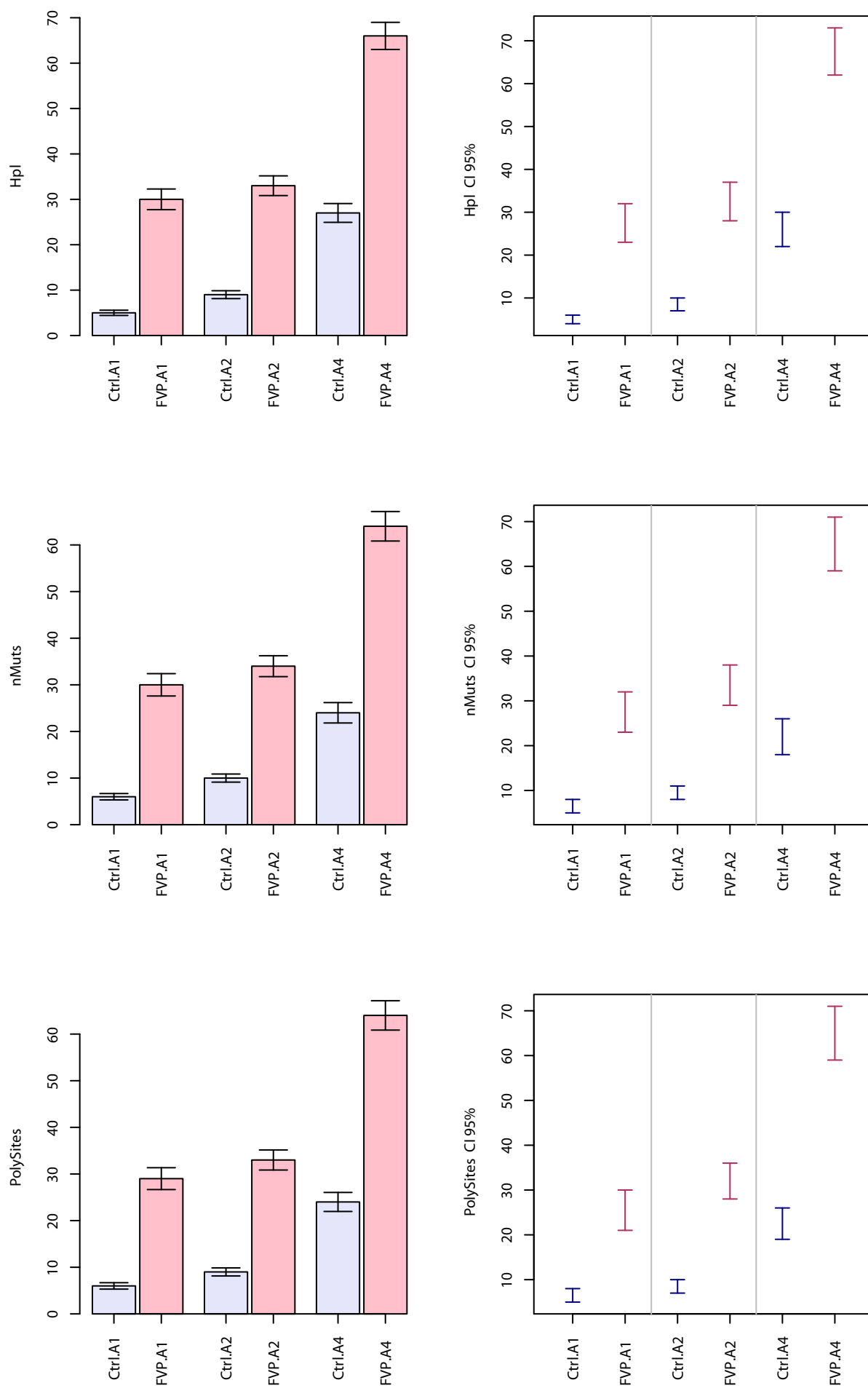

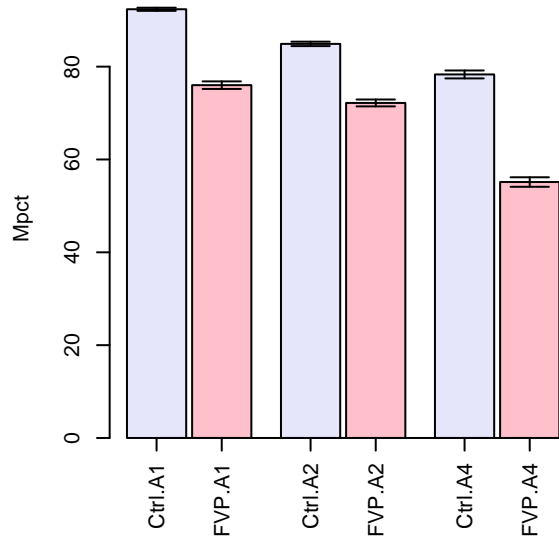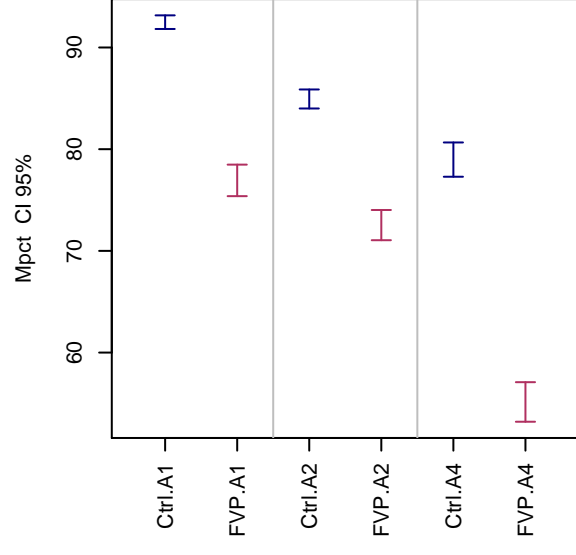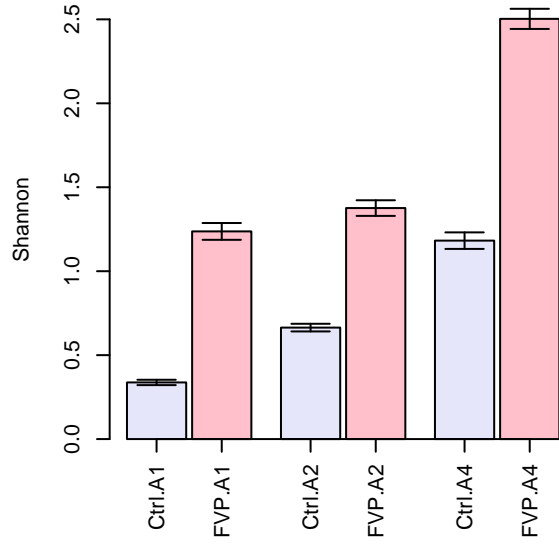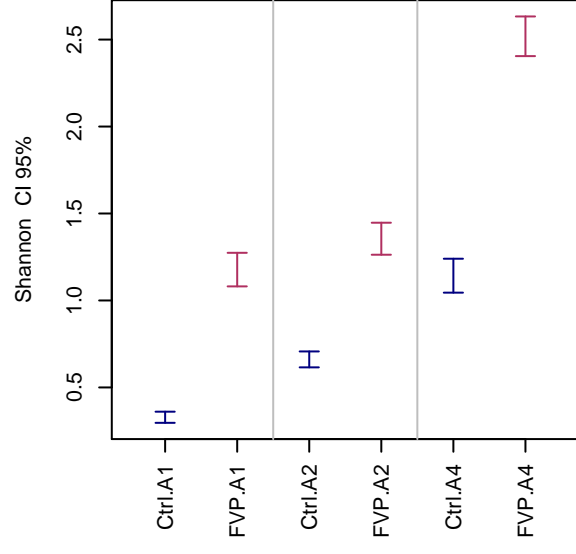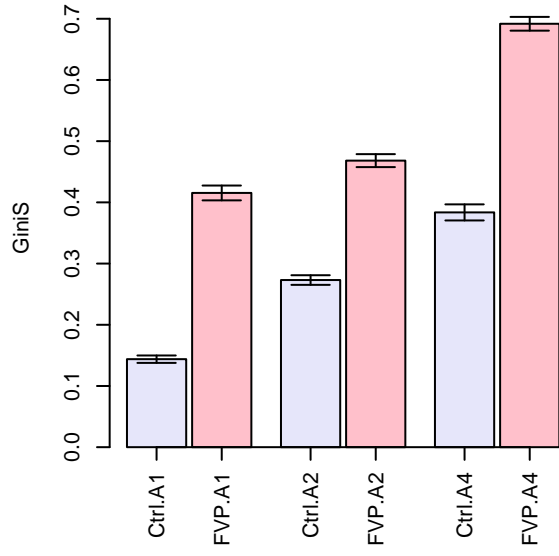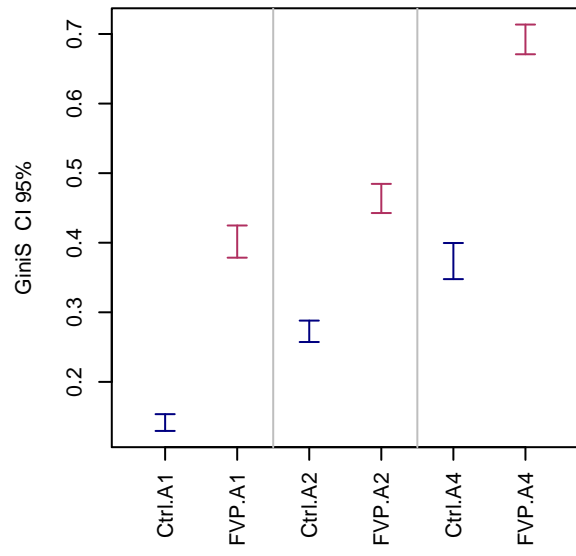

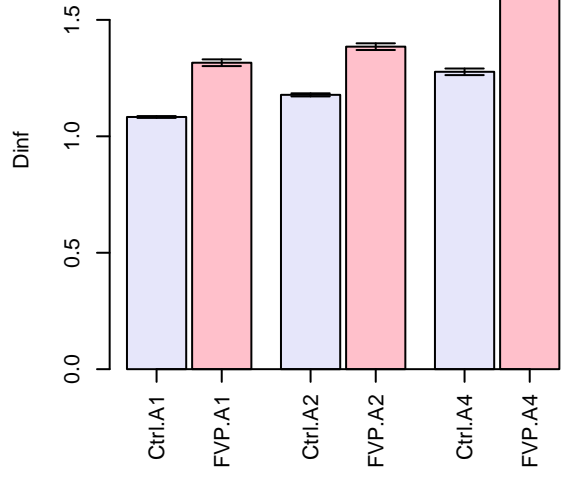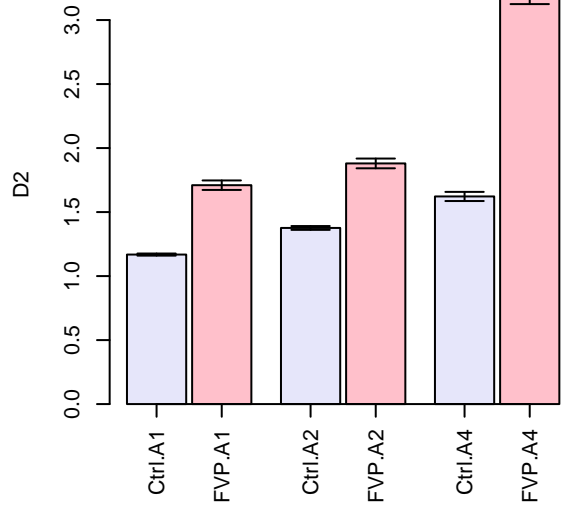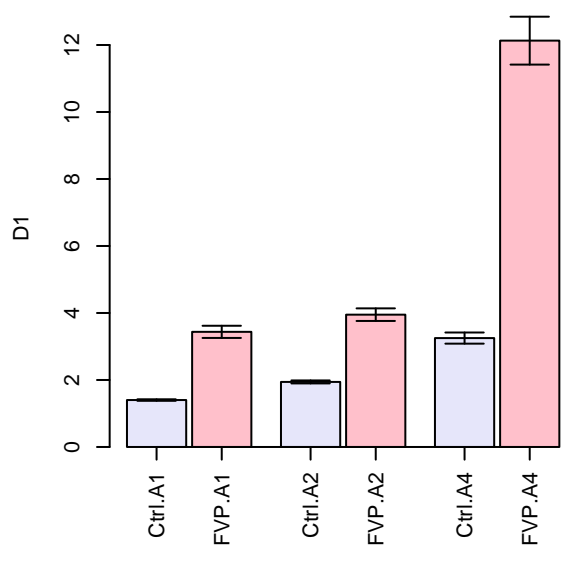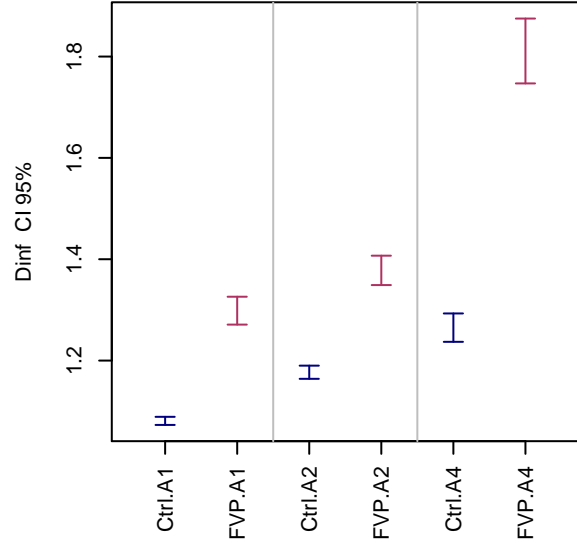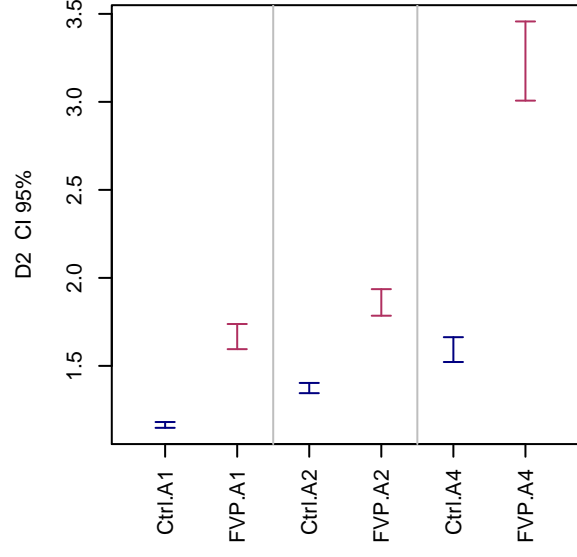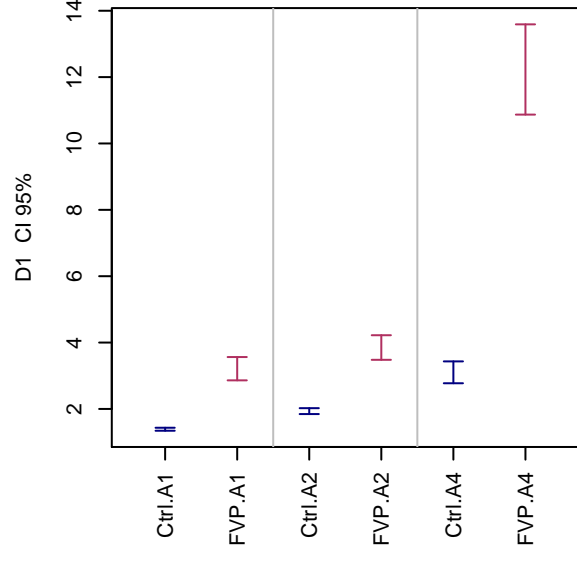

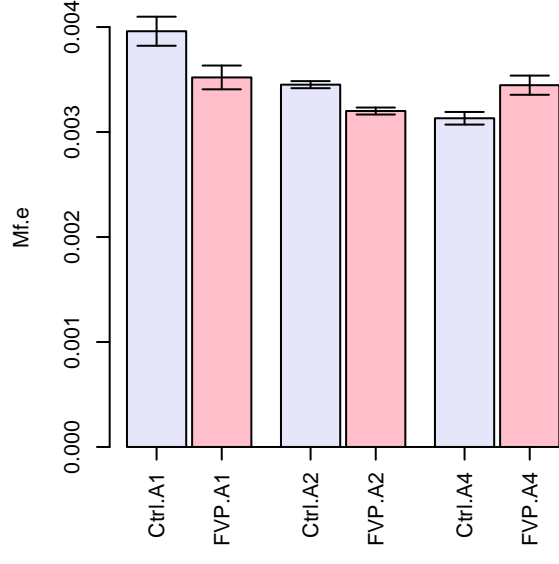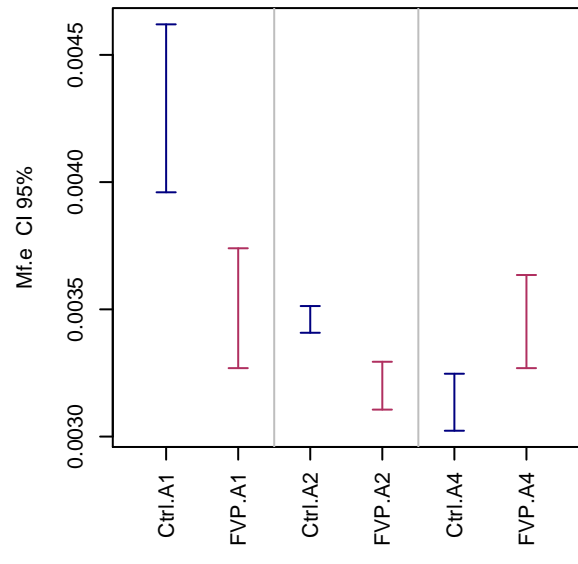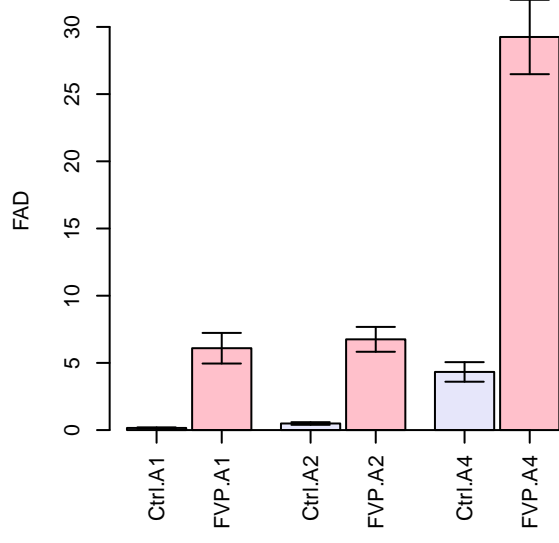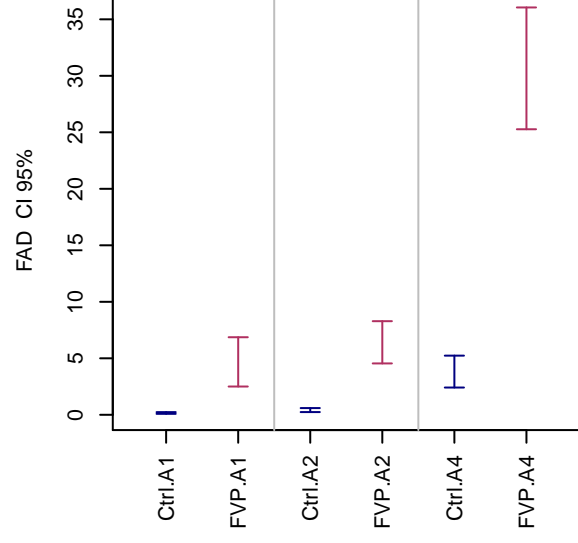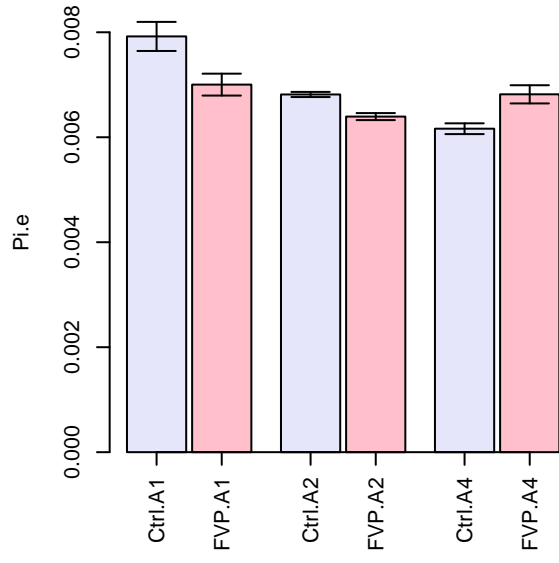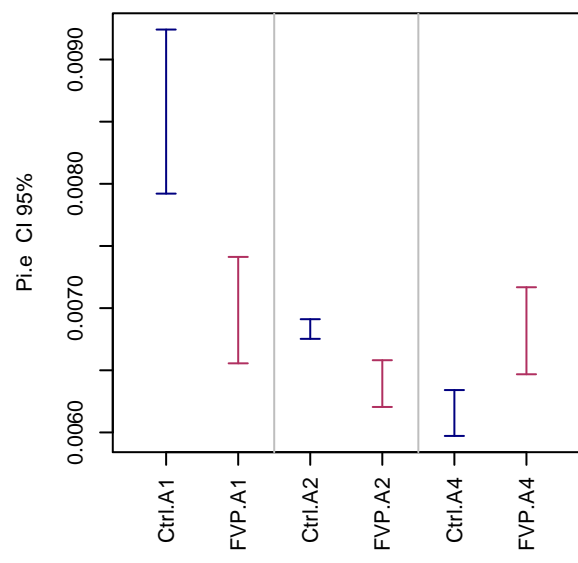

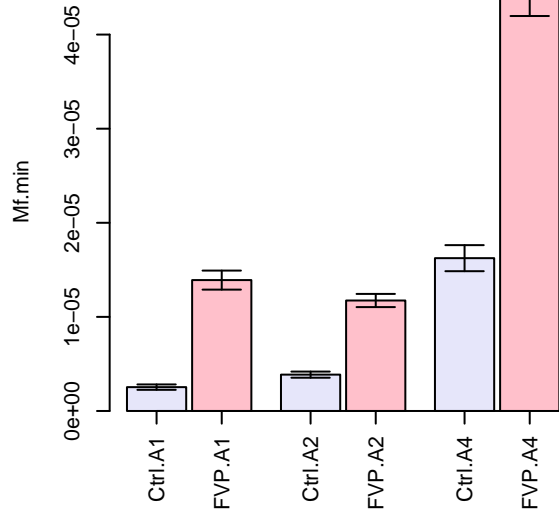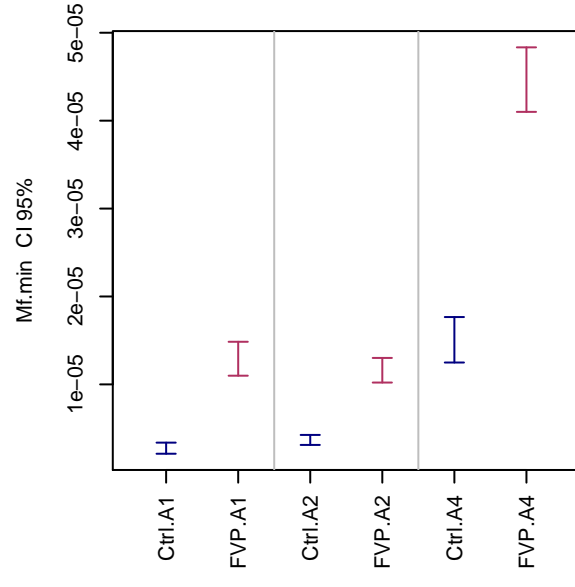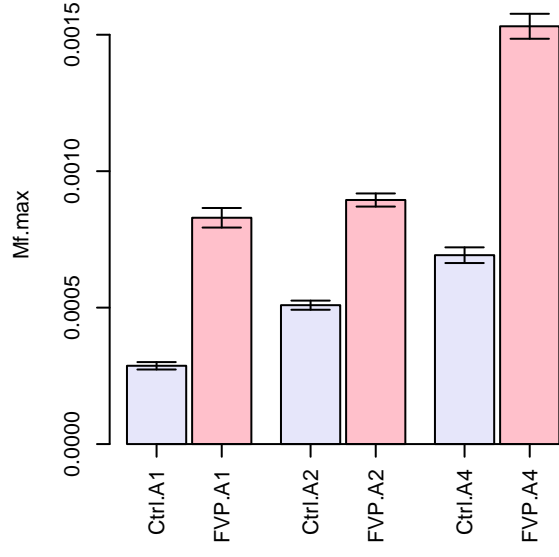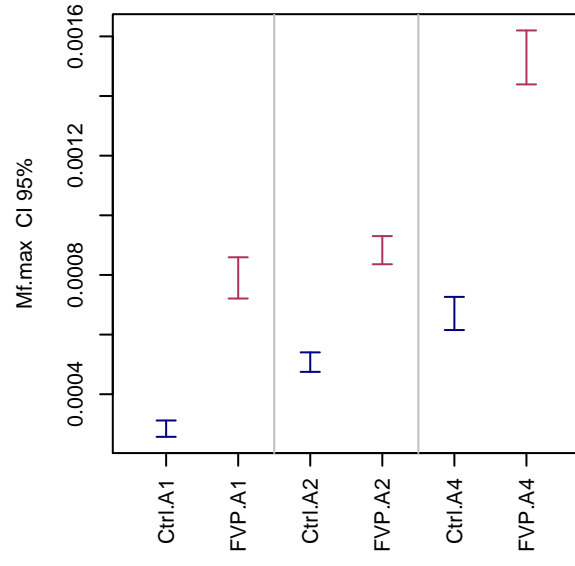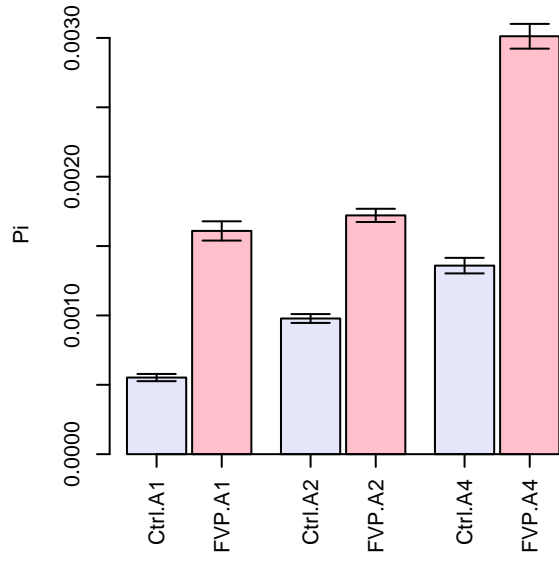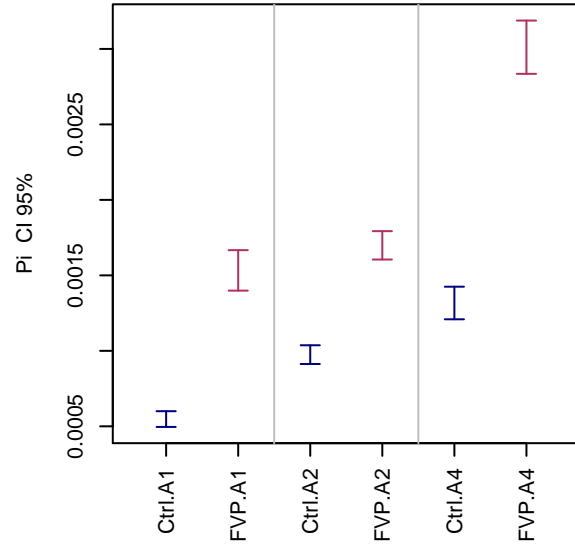

Supplement: S1 Fig — The diversity indices are abbreviated in ordinate (Hpl, number of haplotypes; nMuts, number of different mutations; PolySites, number of polymorfic sites; Mpct; dominant haplotype abundance; Shannon, ^HS; GiniS, ^HGS, sample-based Gini-Simpson index; D1, D2, Dinf, Hill numbers; Mfe, mutation frequency, entity level; FAD, Functional Attribute Diversity; Pi.e, sample nucleotide diversity, entity level; Mf min, minimum mutation frequency; Mf.max, maximum mutation frequency; Pi, sample nucleotide diversity, and their calculation is described in reference [47] of the main text. Standard deviation interval (left column), and basic bootstrap with 95% confidence intervals (CI)(right column) are shown for each index. (PDF) [file pone.0164691.s001.pdf]

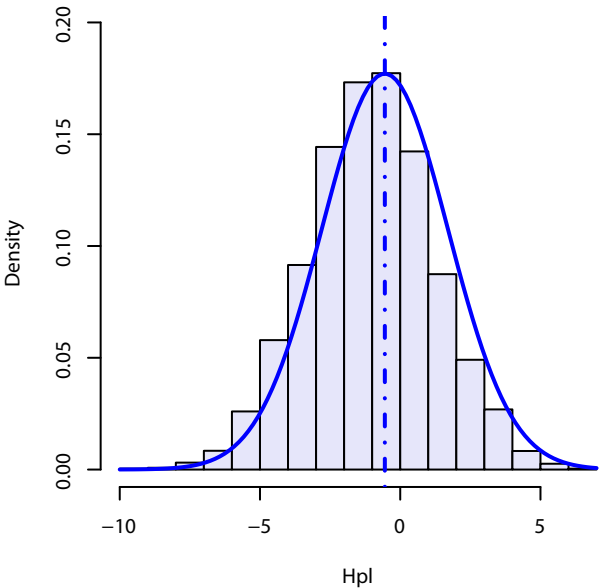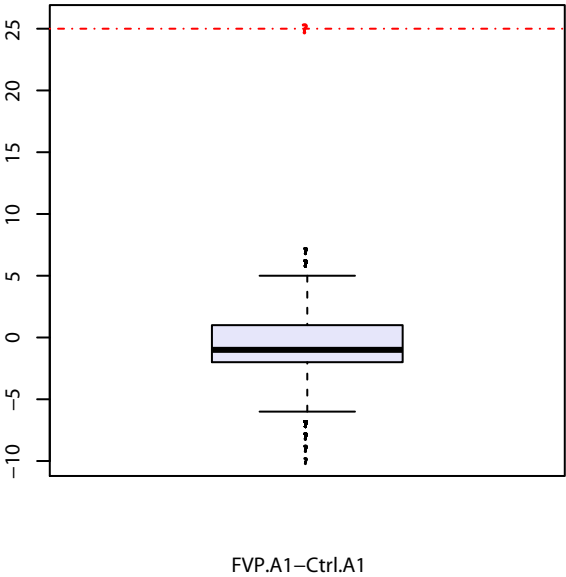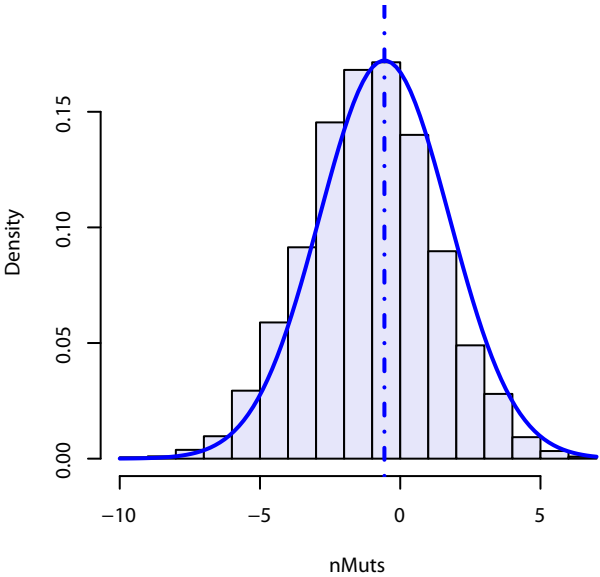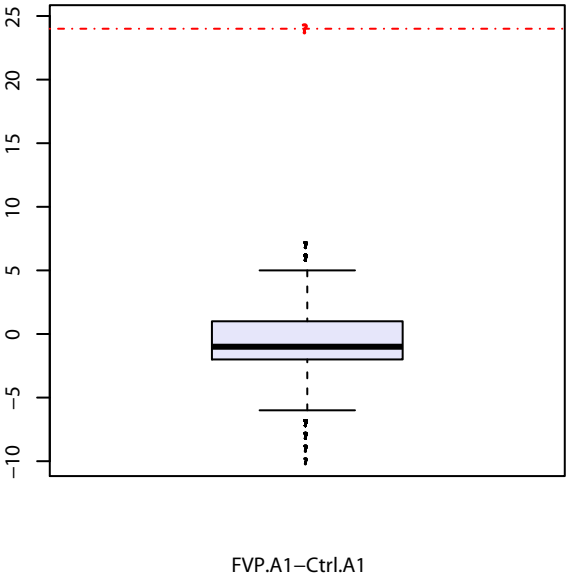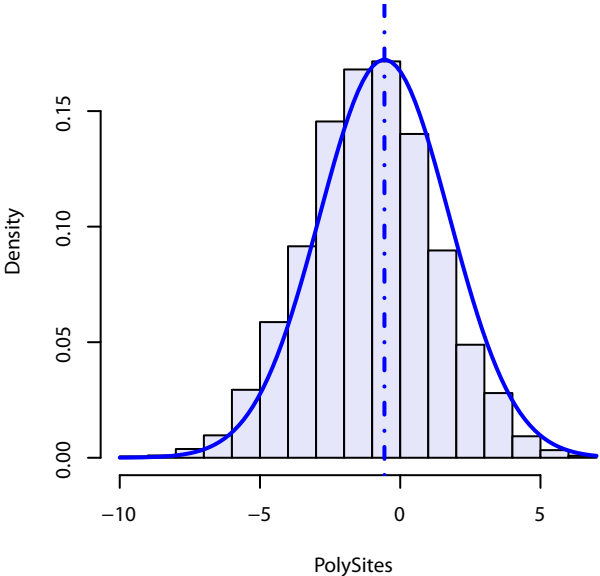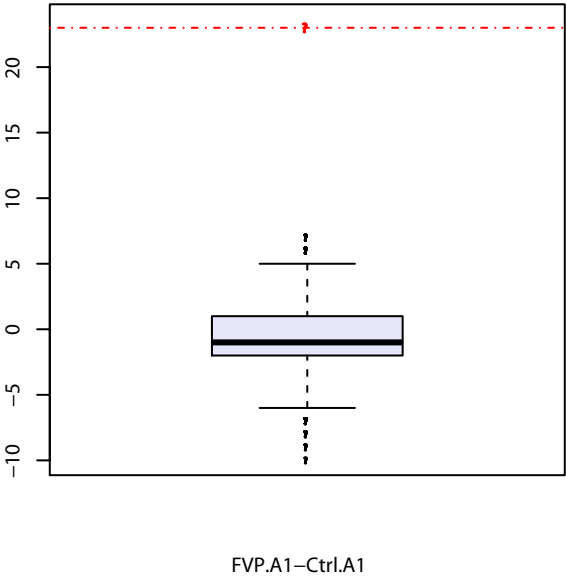

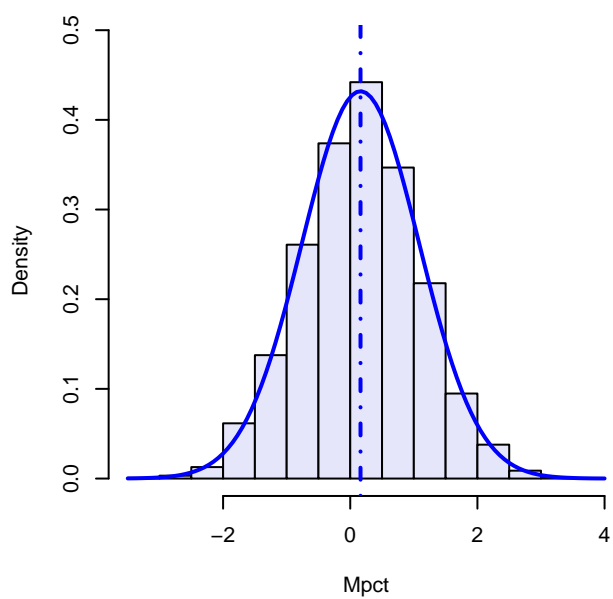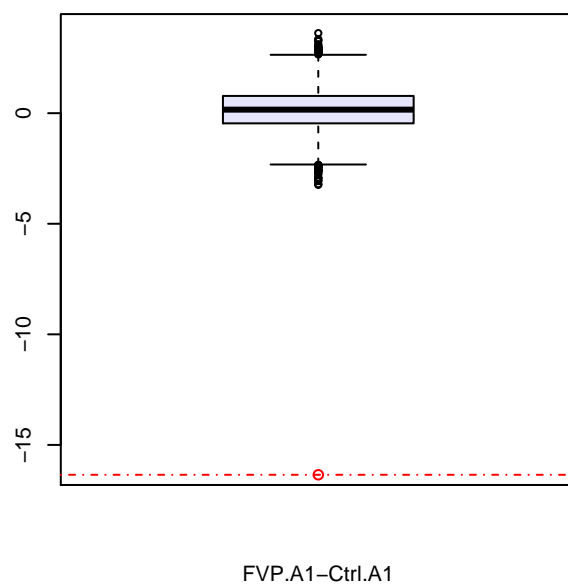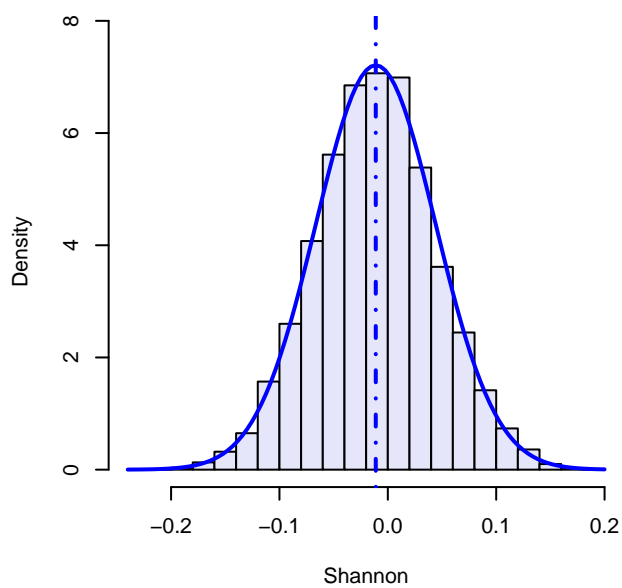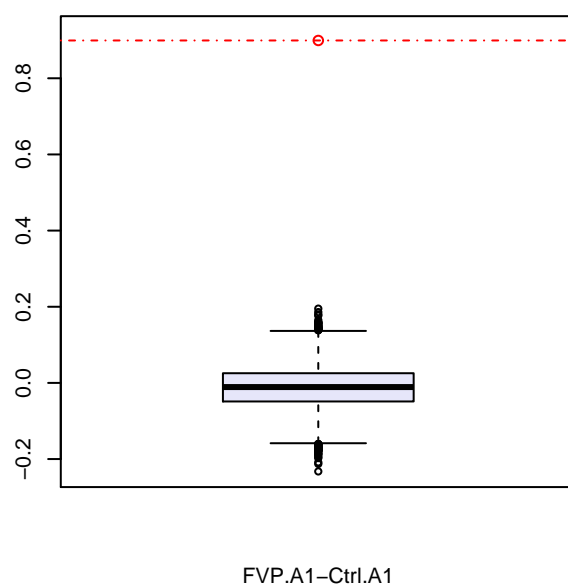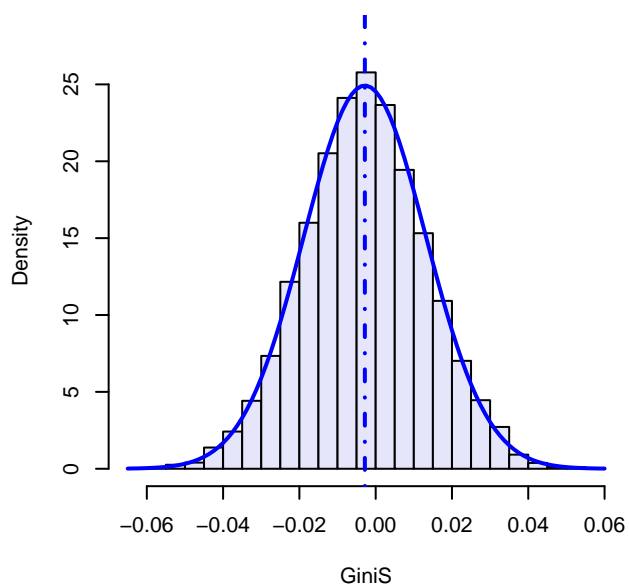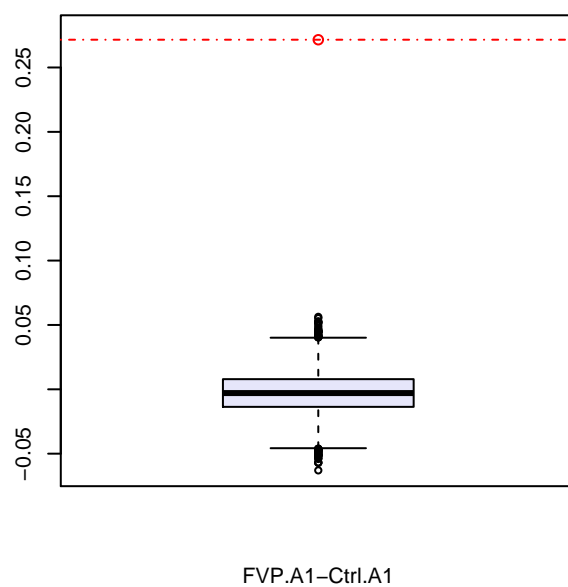

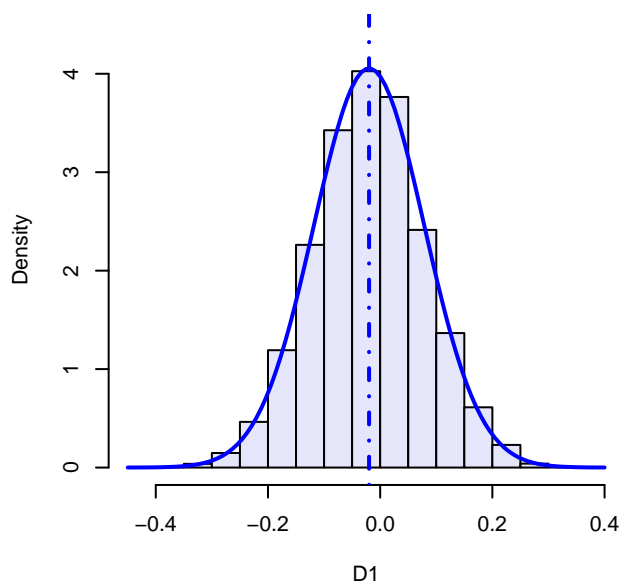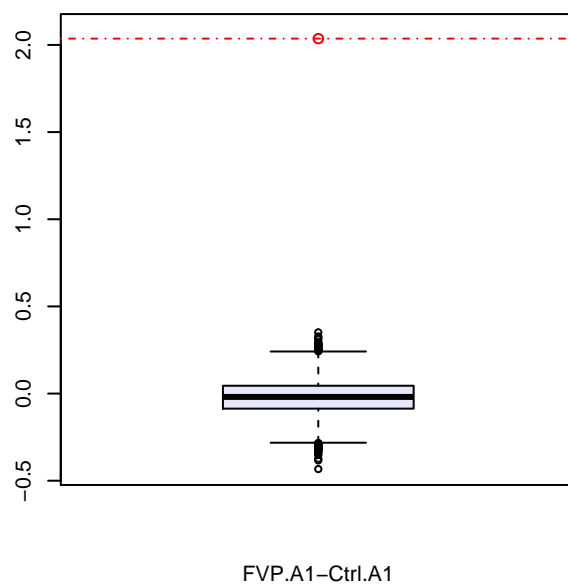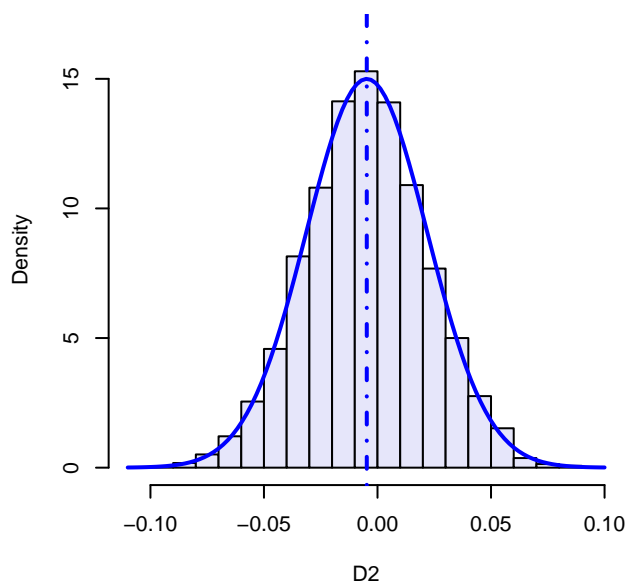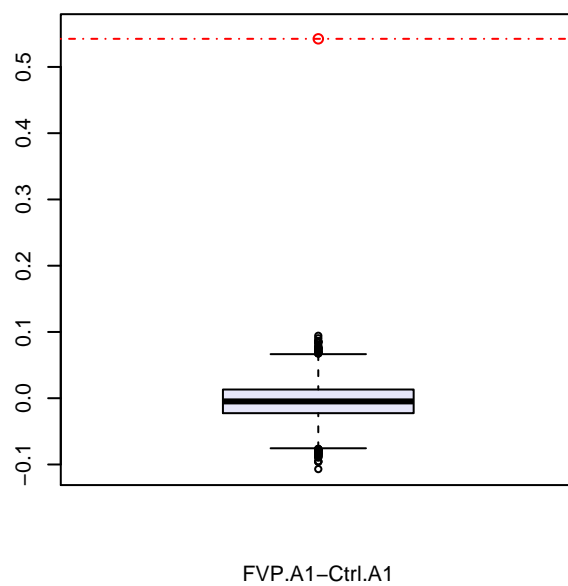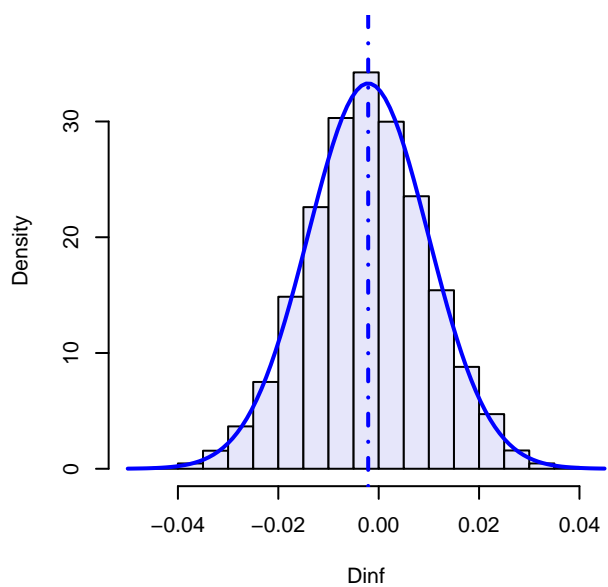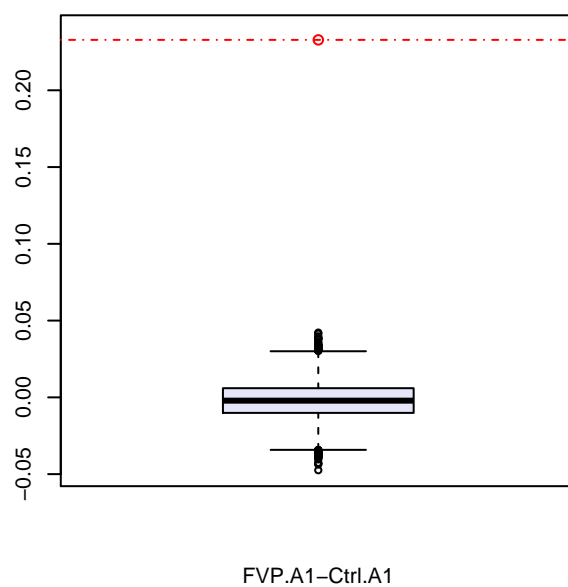

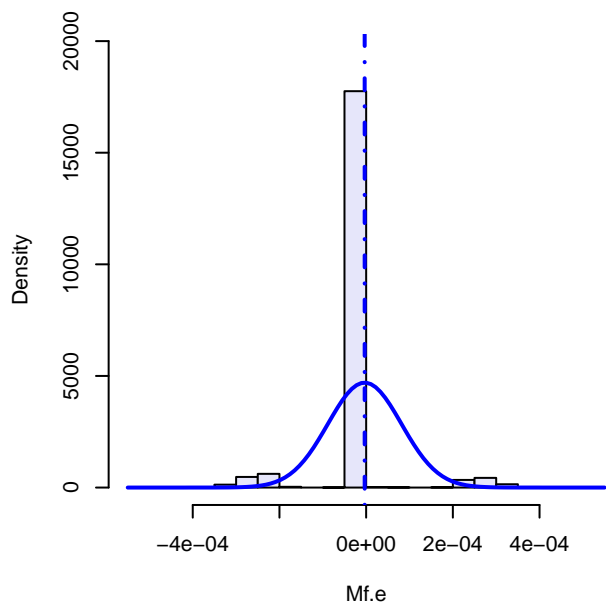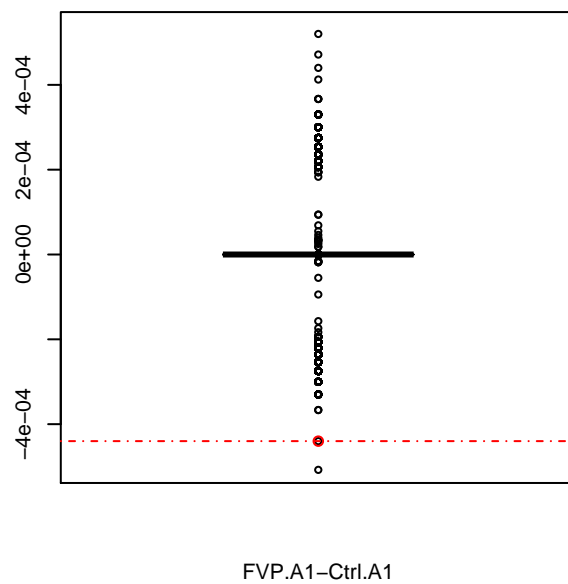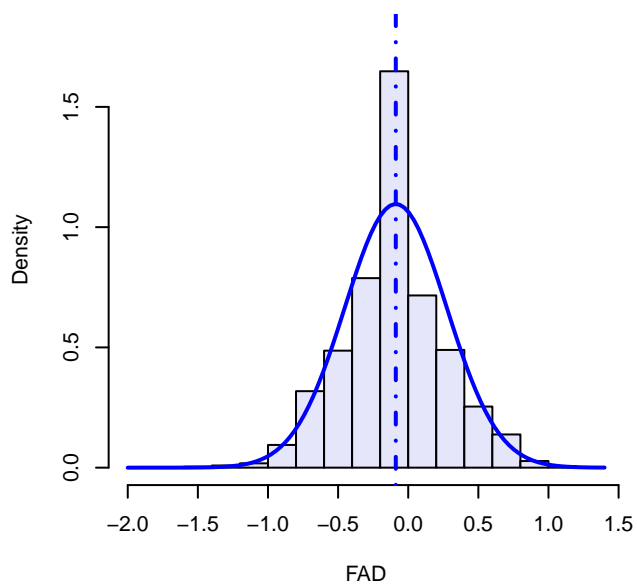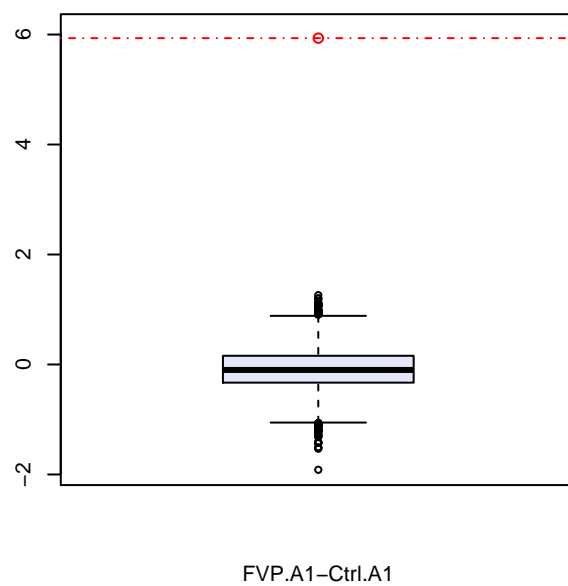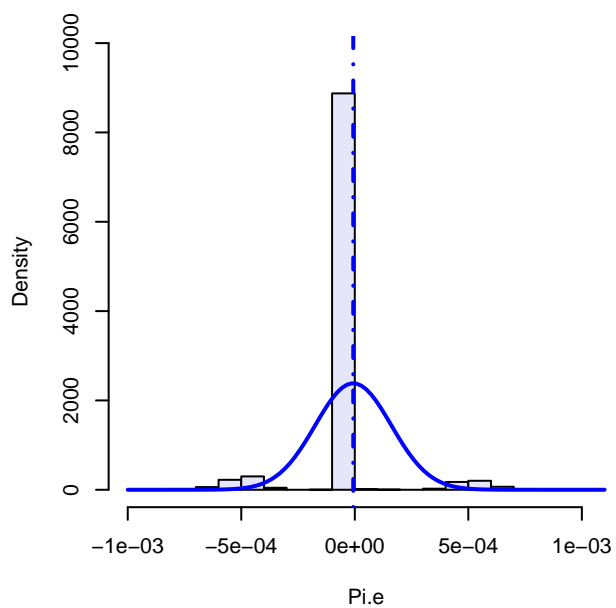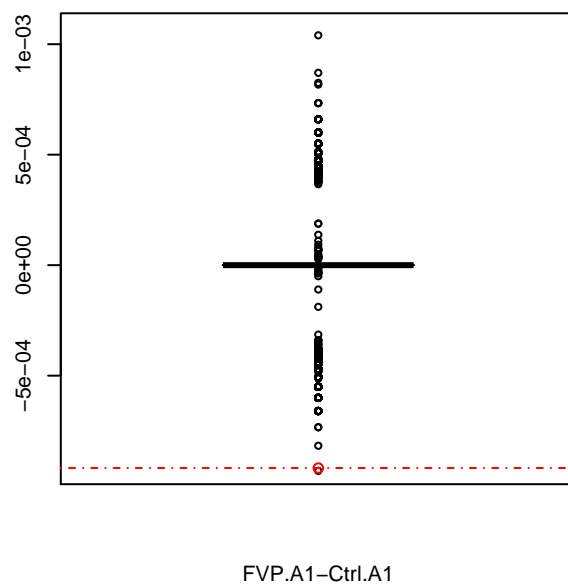

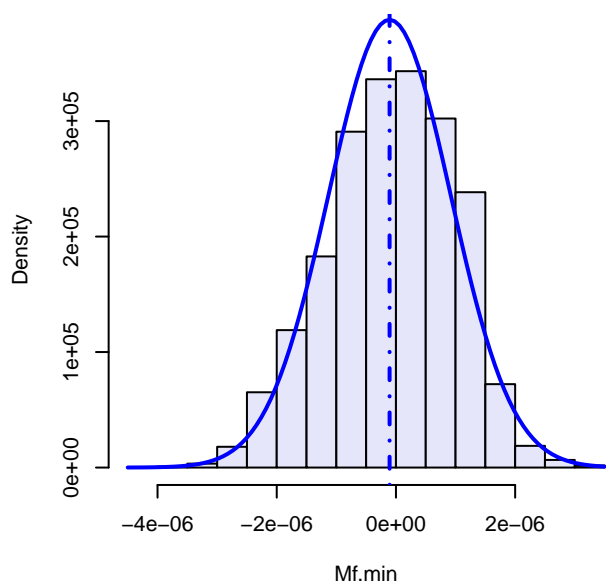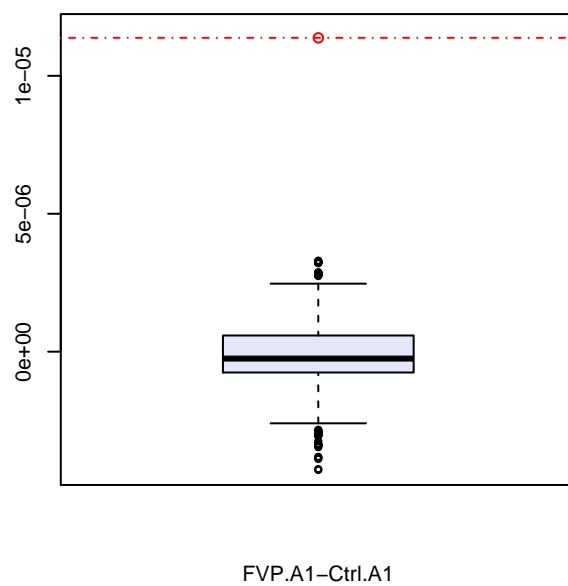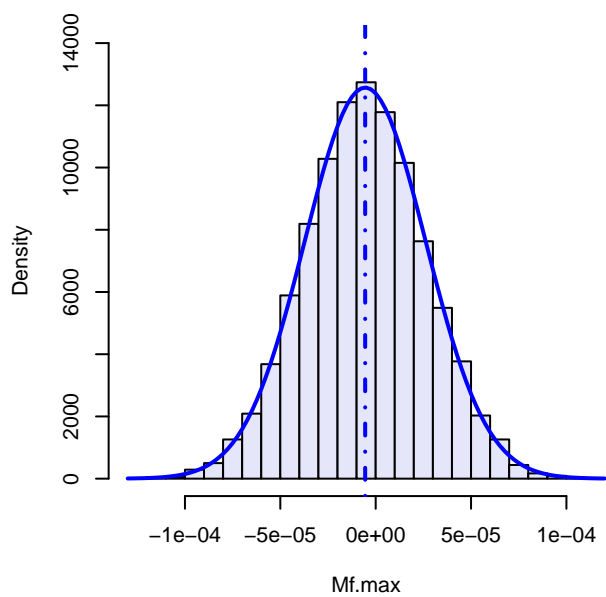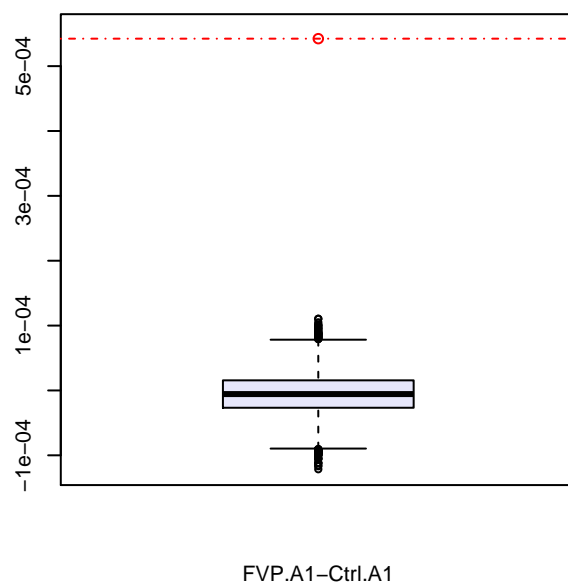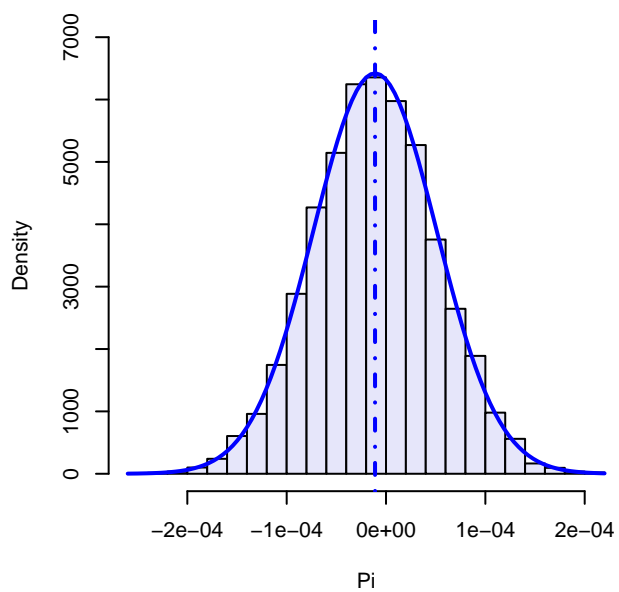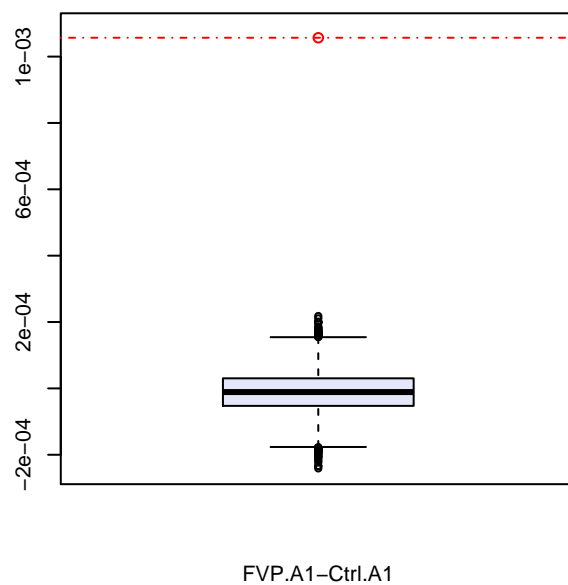

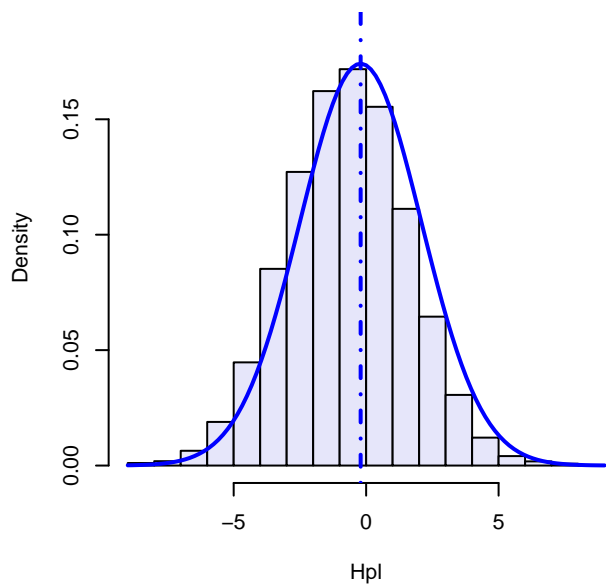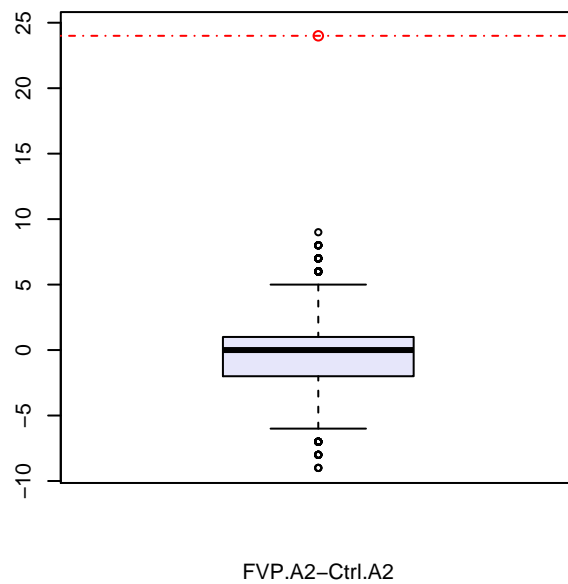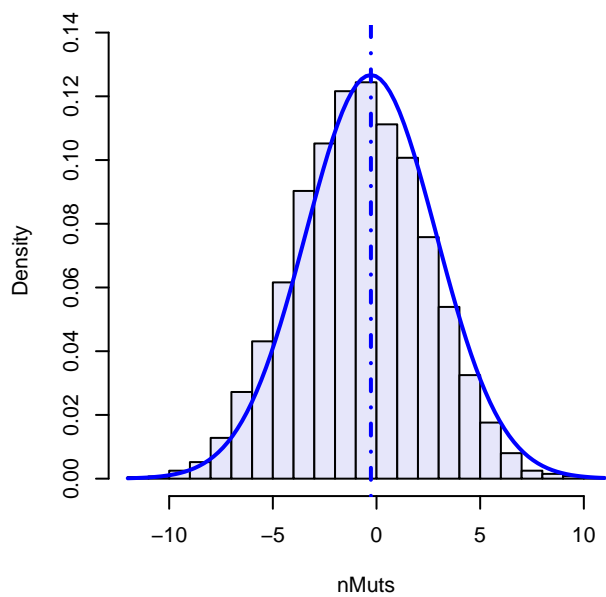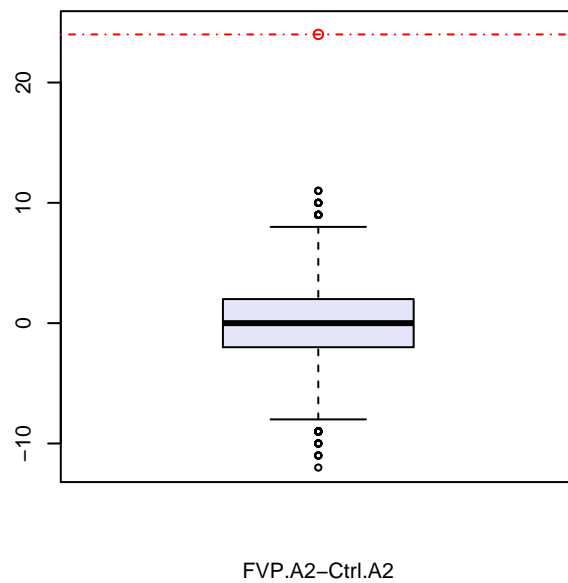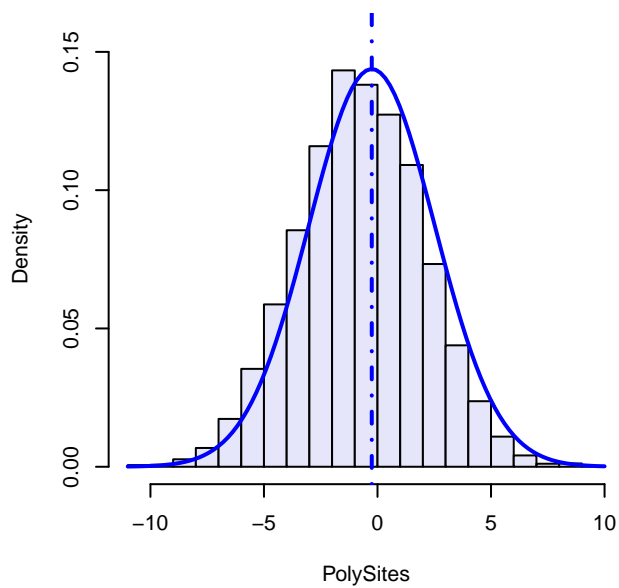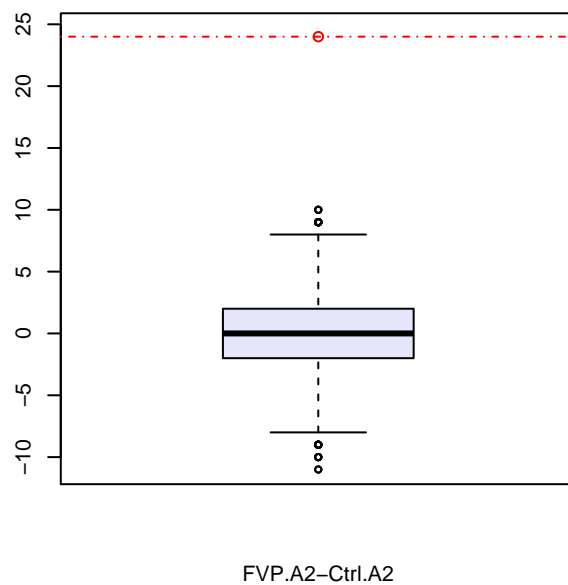

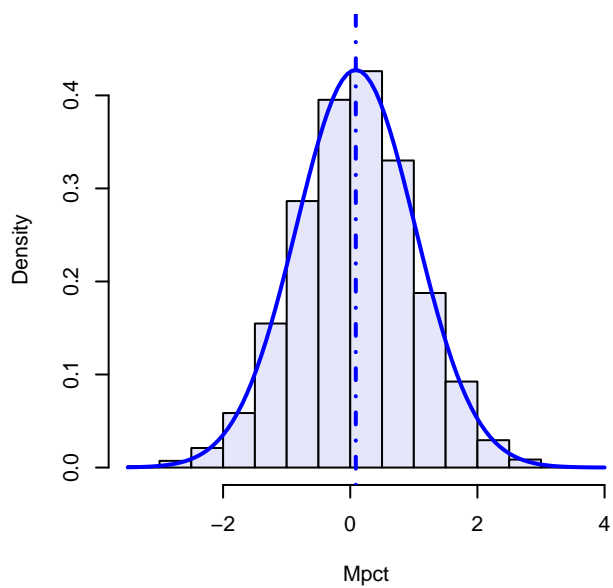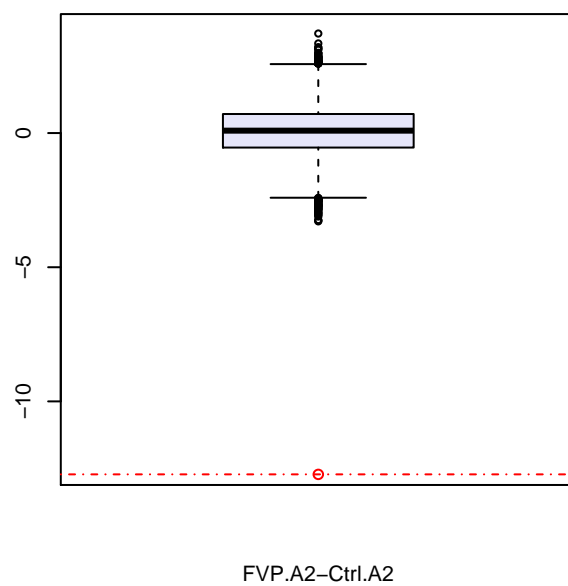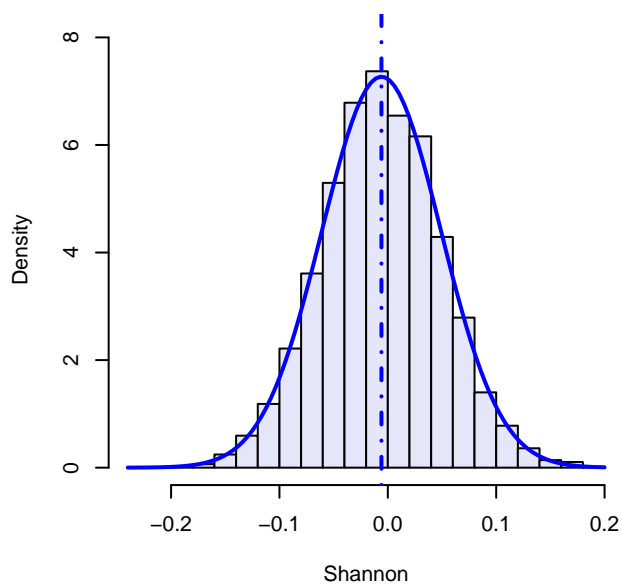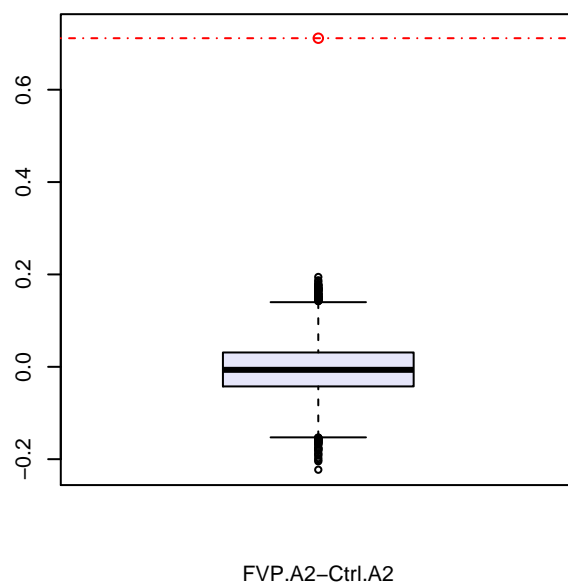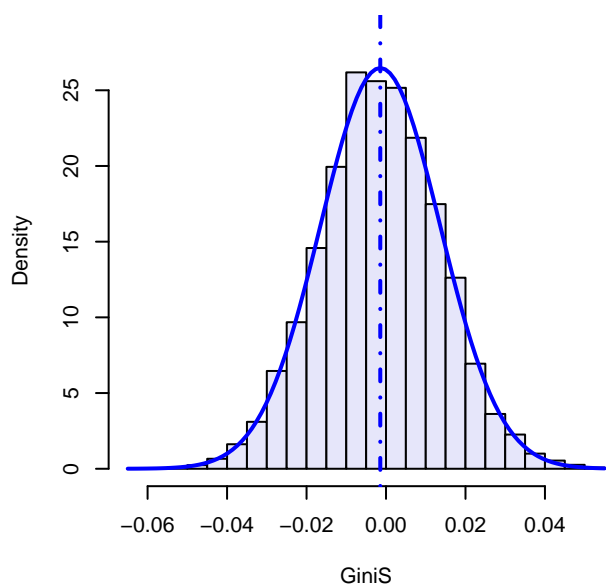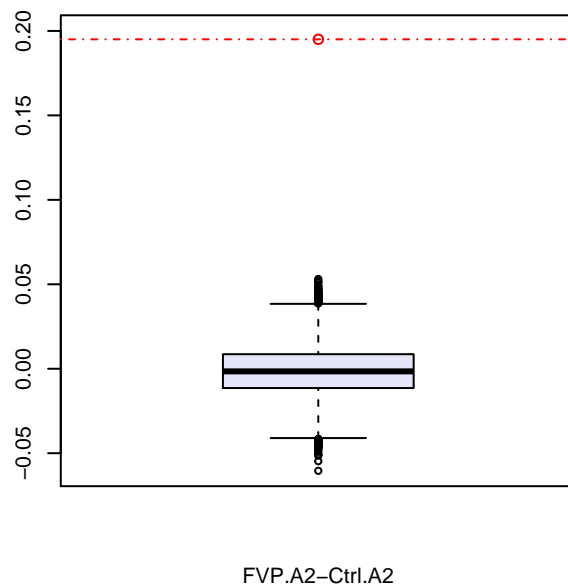

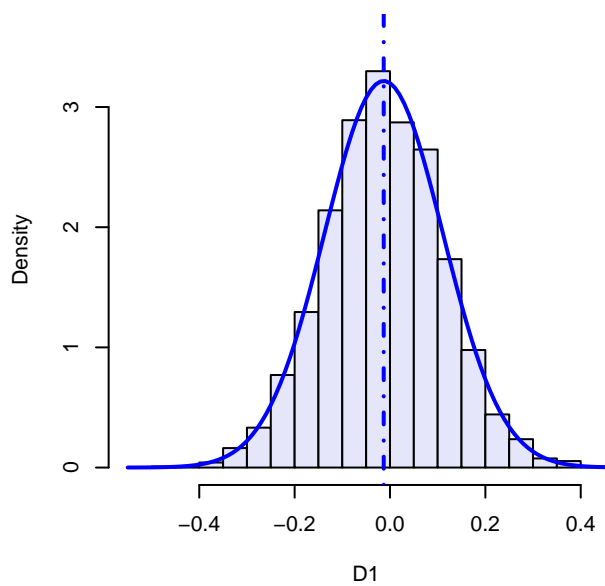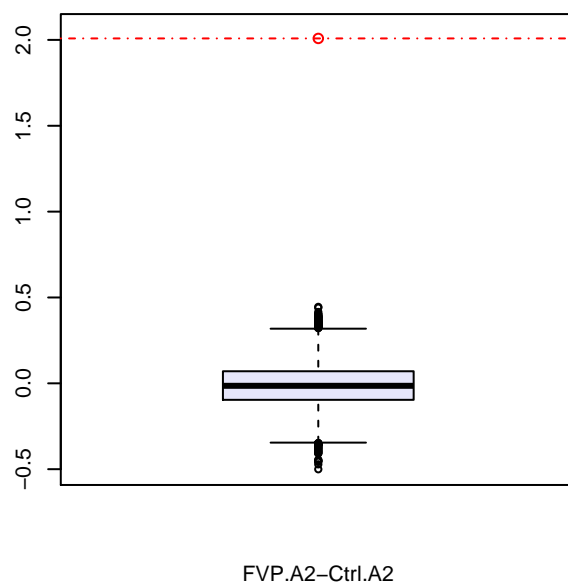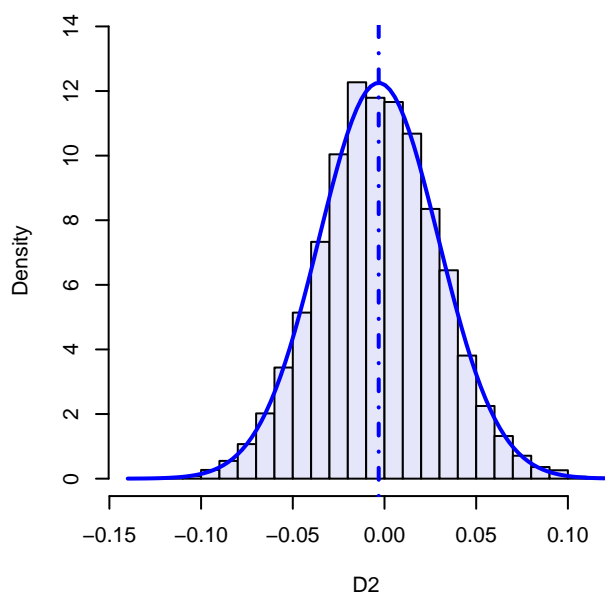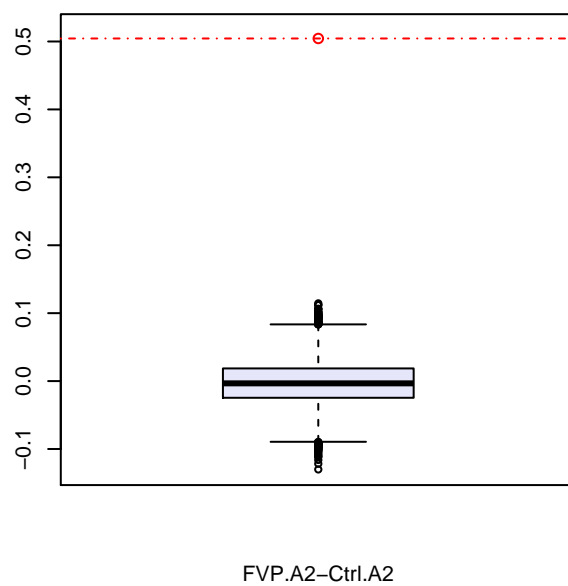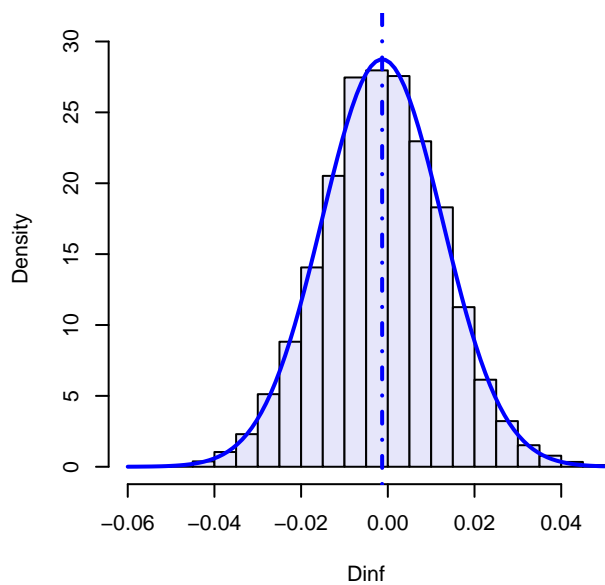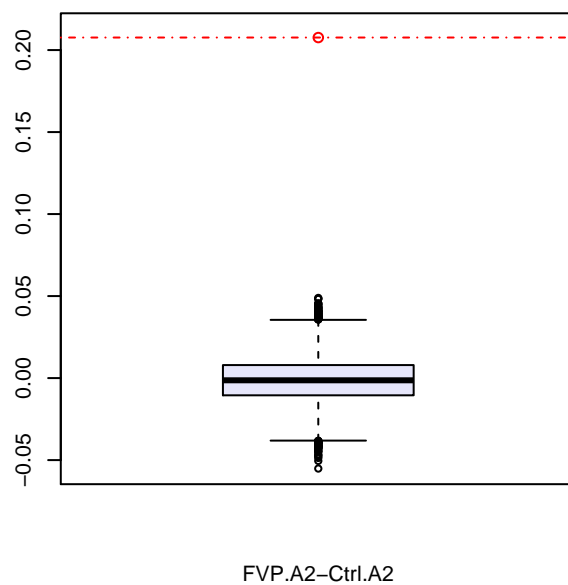

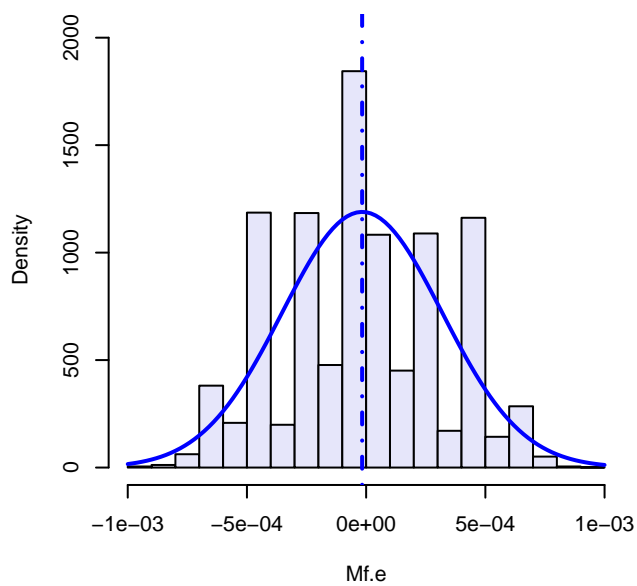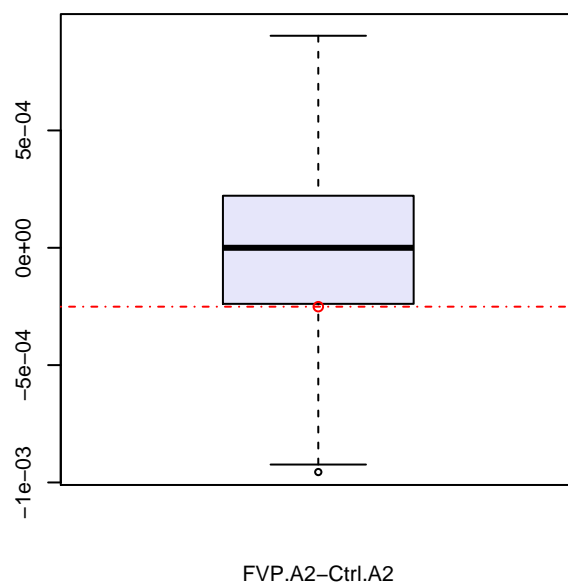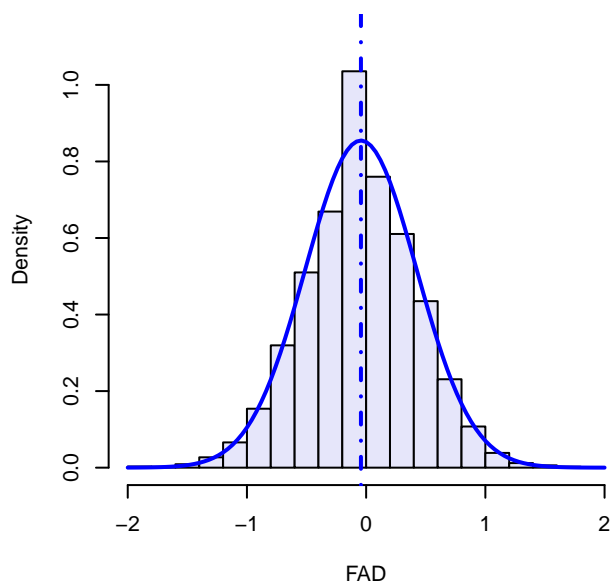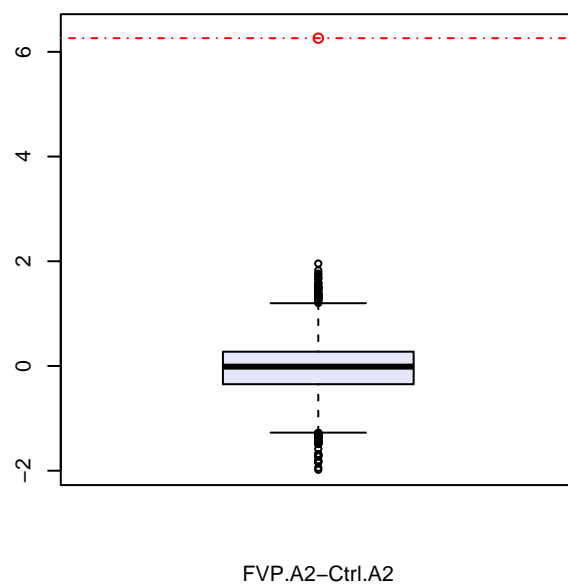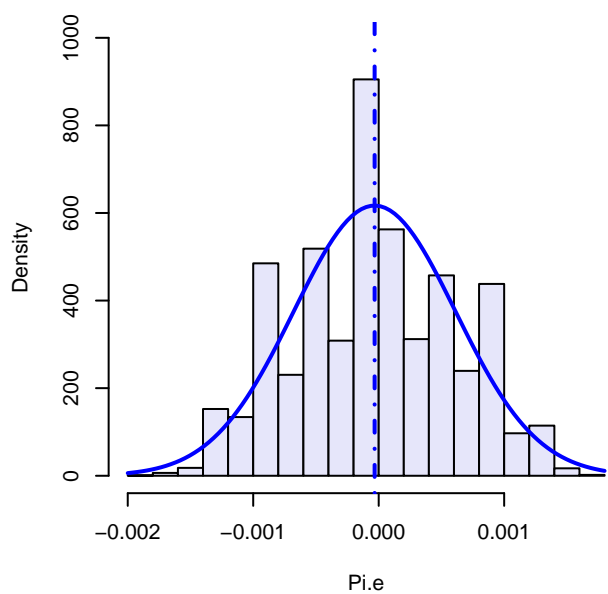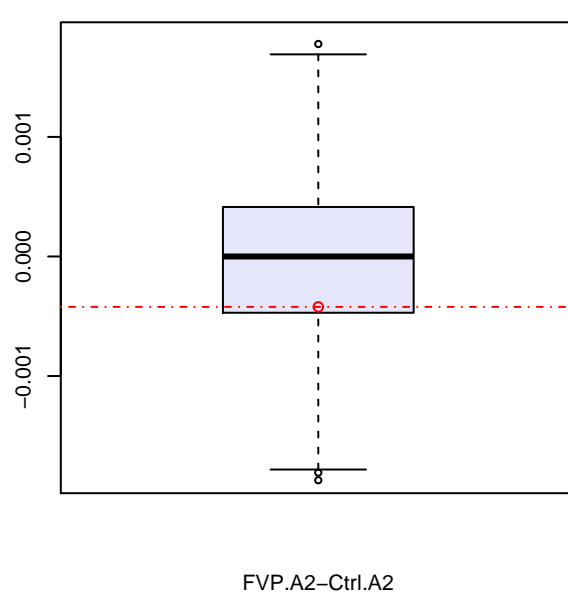

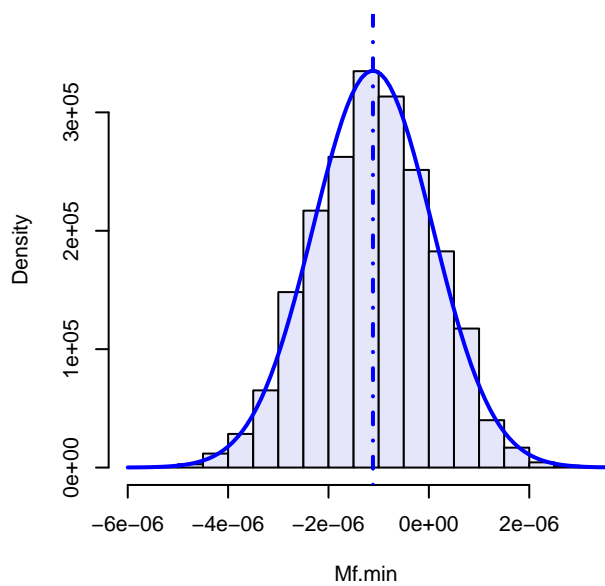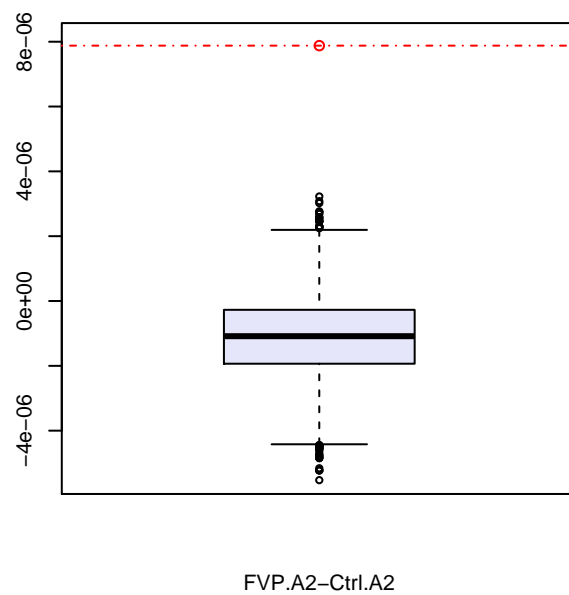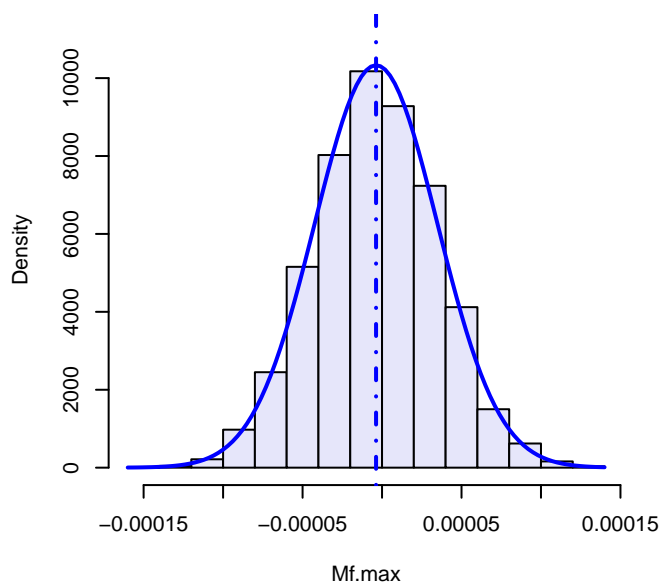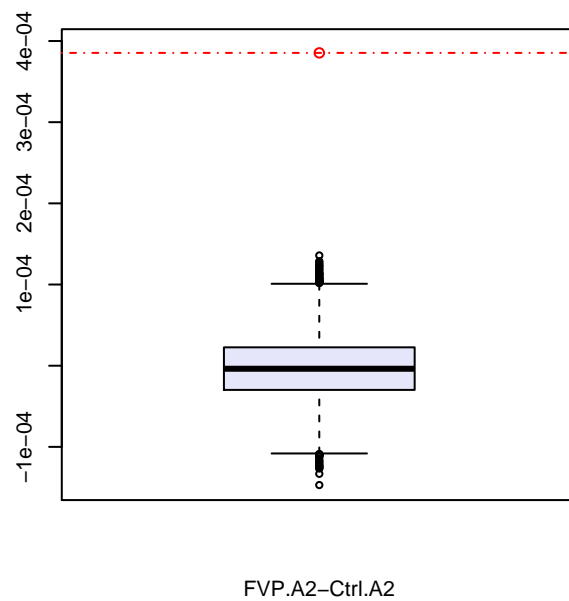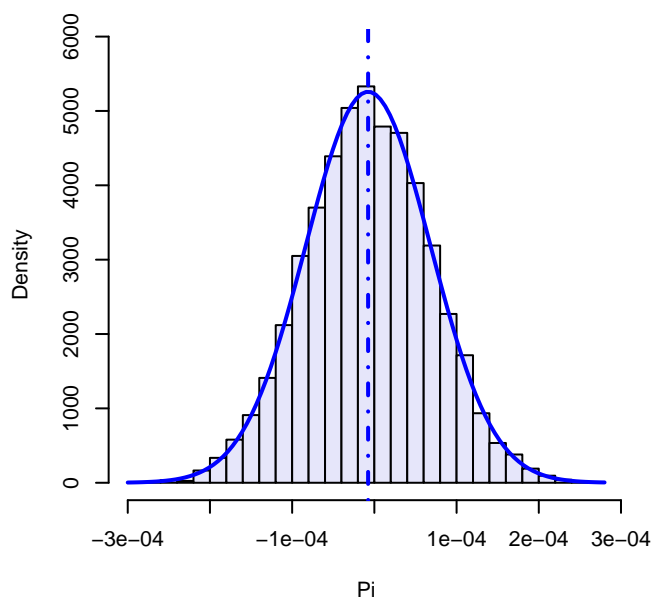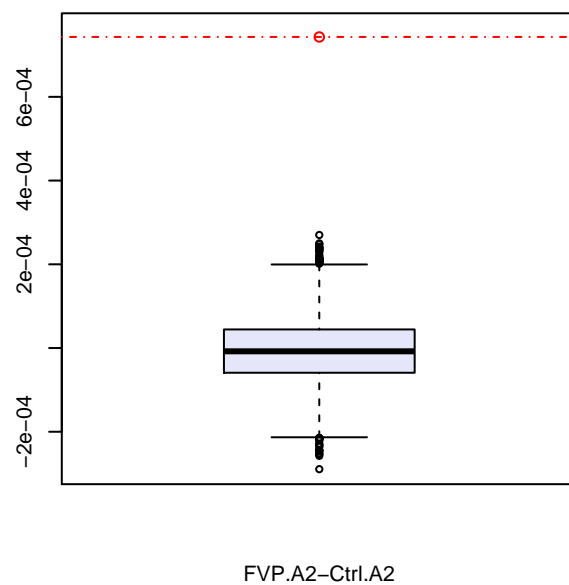

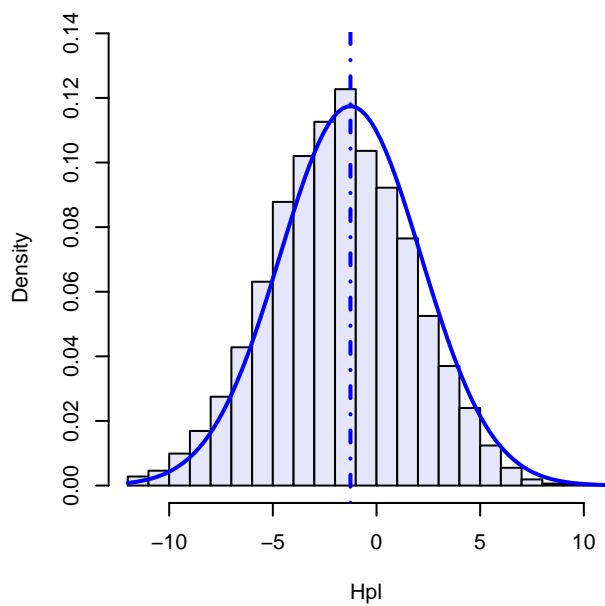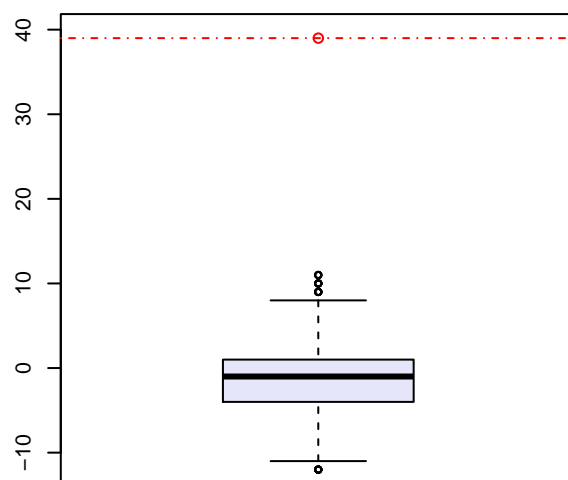

FVP.A4-Ctrl.A4

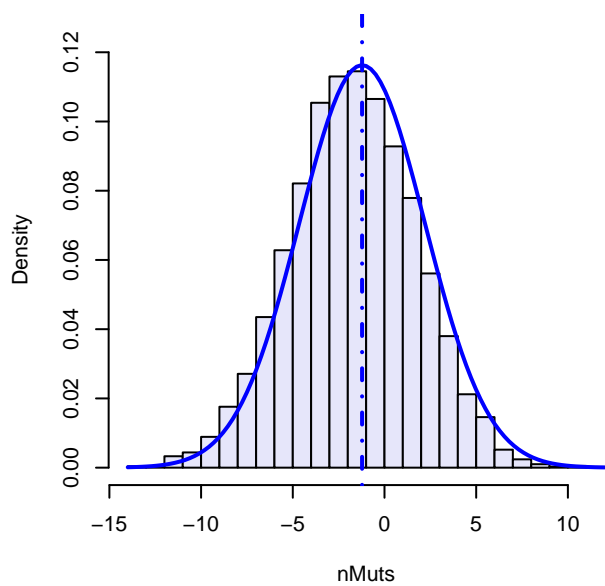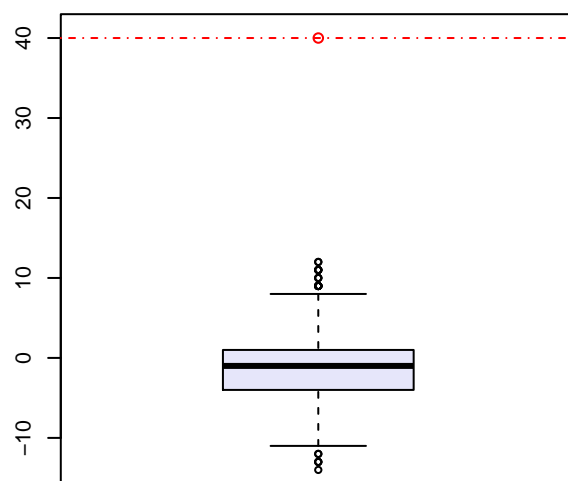

FVP.A4-Ctrl.A4

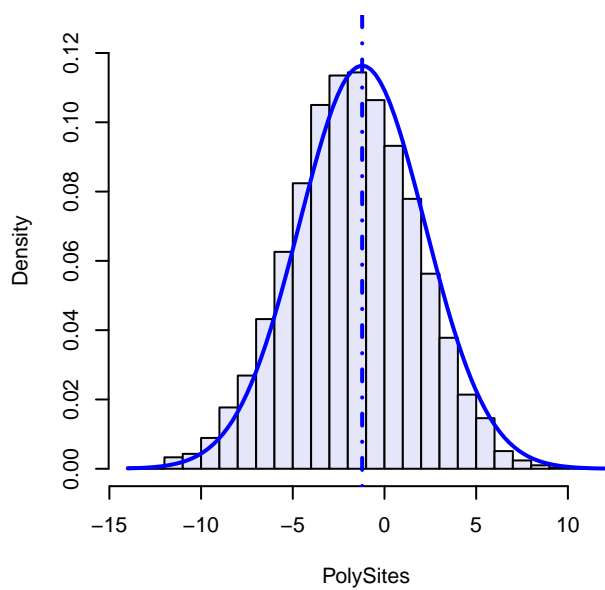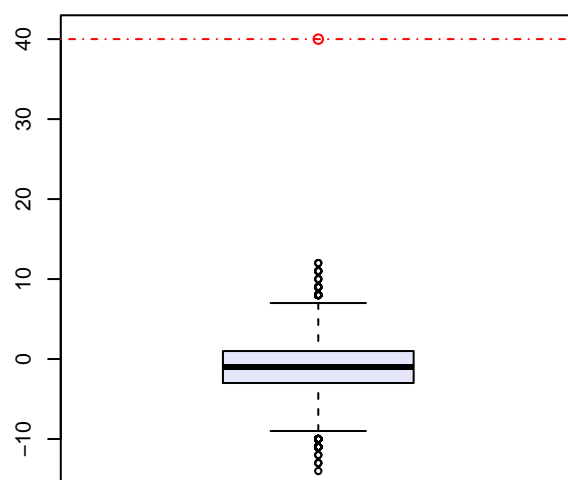

FVP.A4-Ctrl.A4

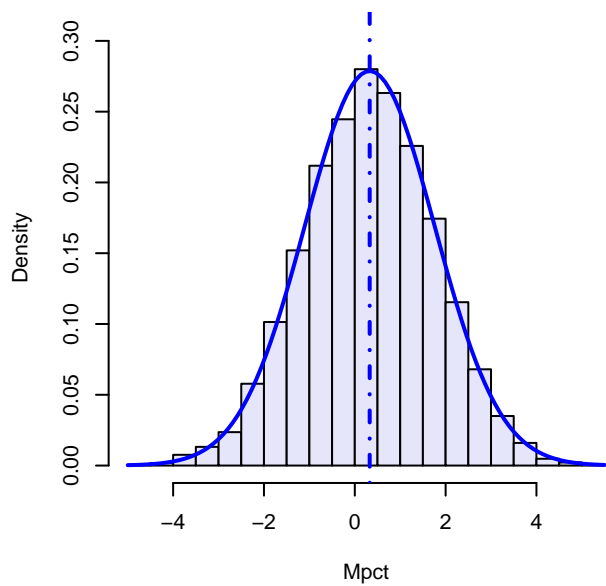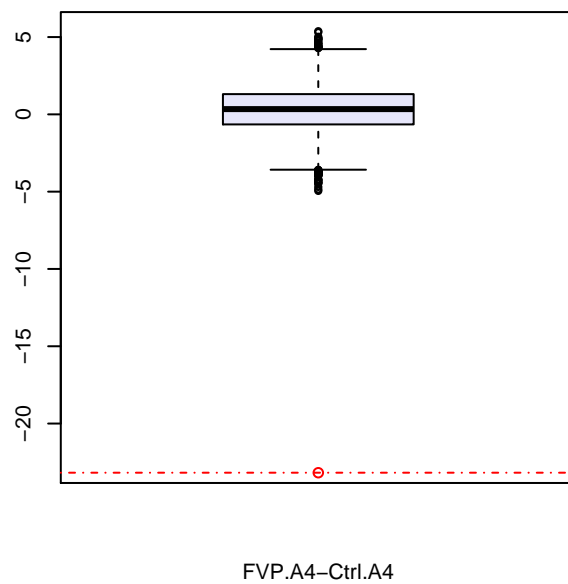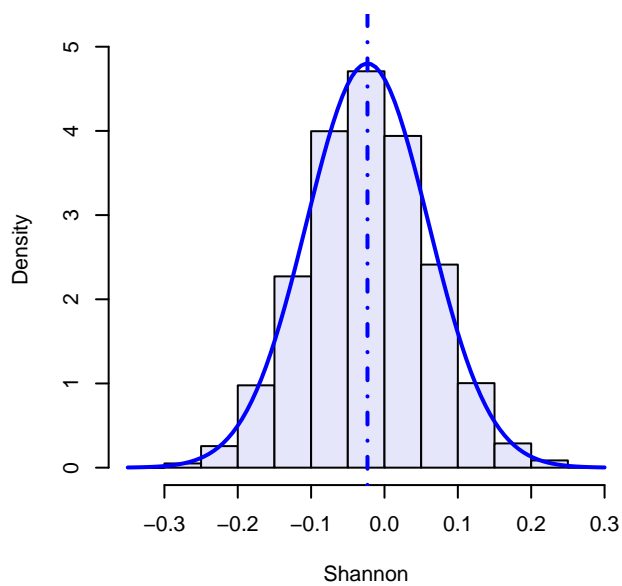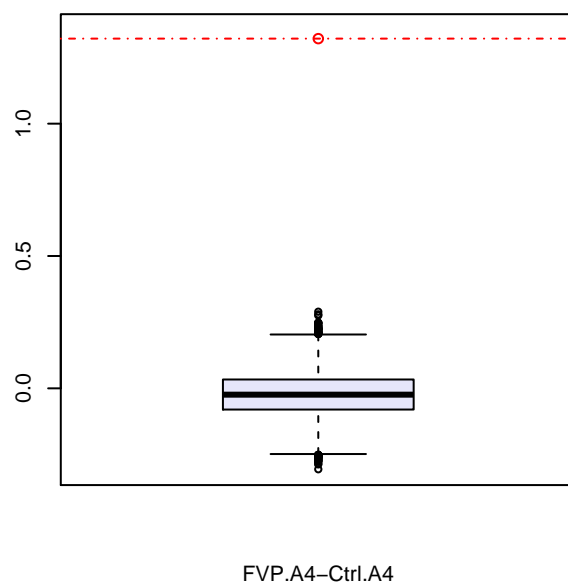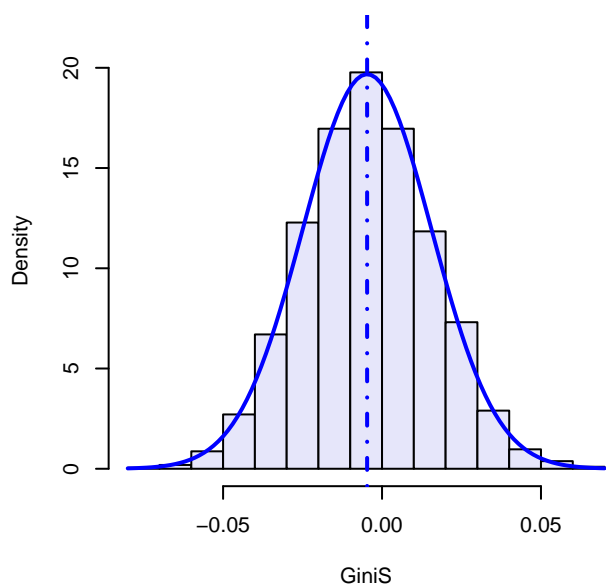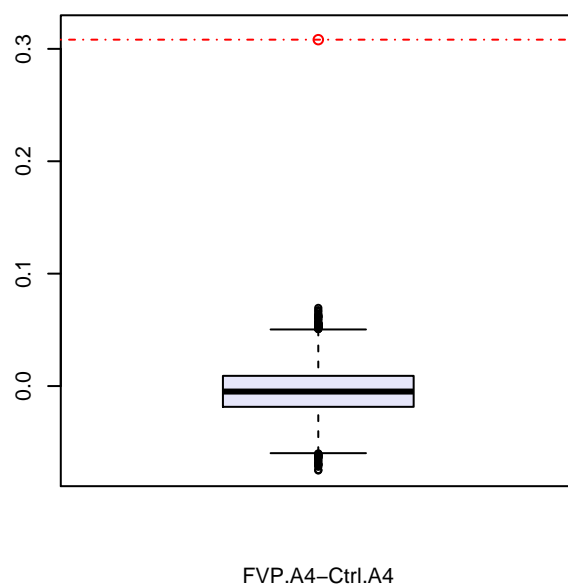

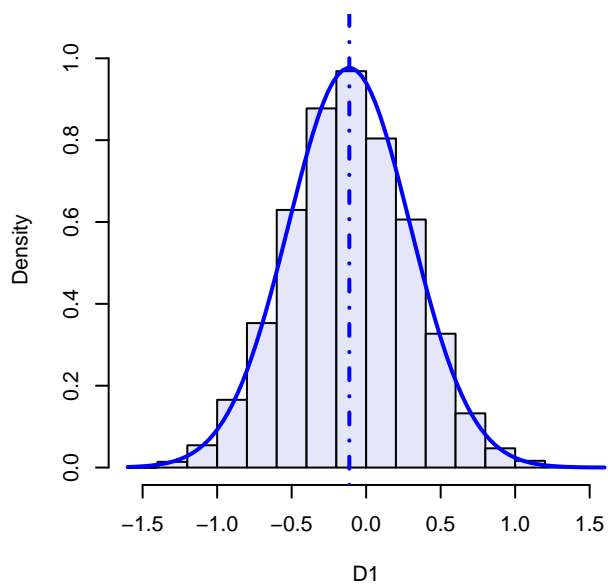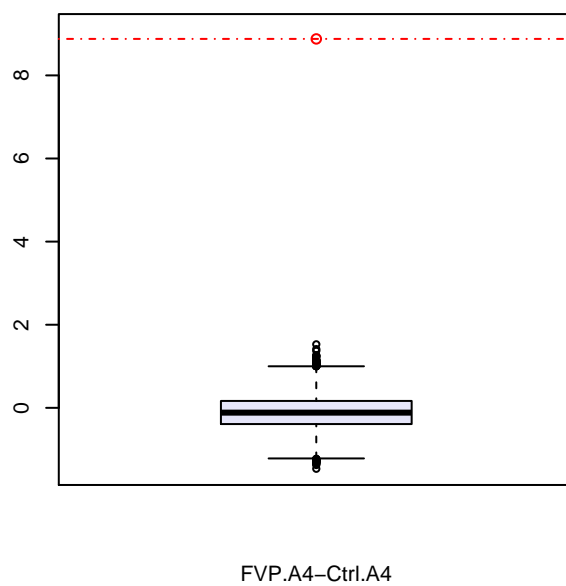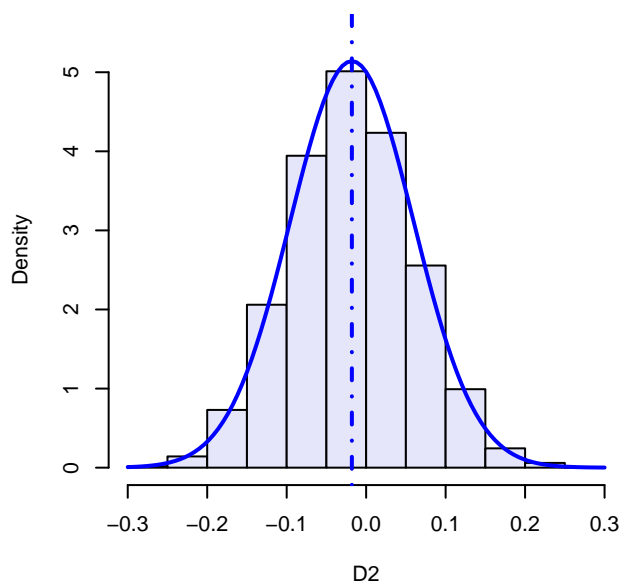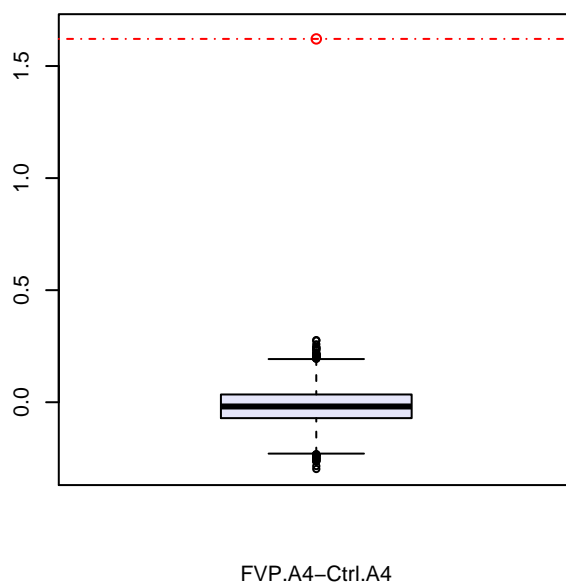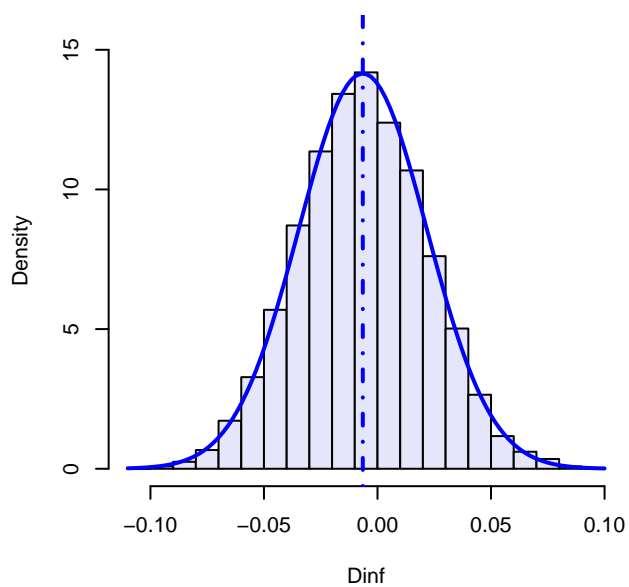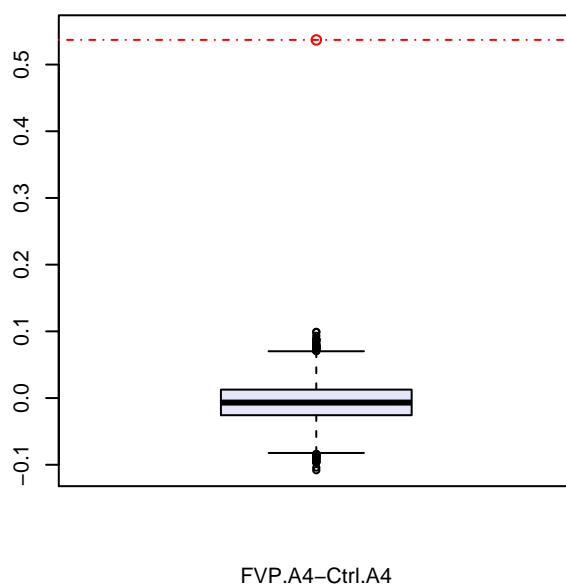

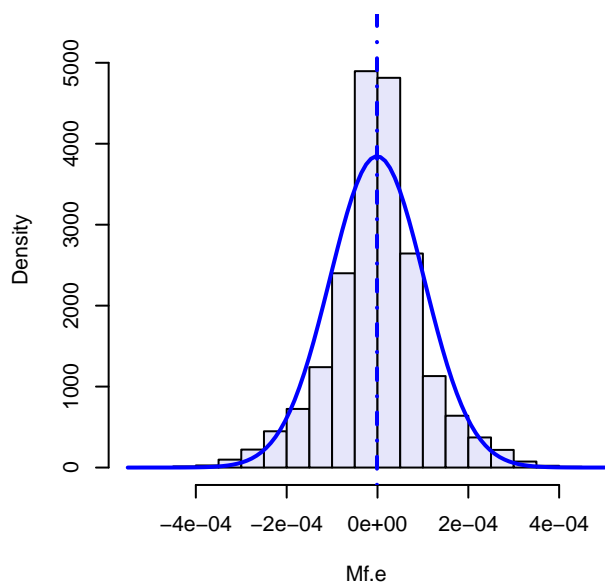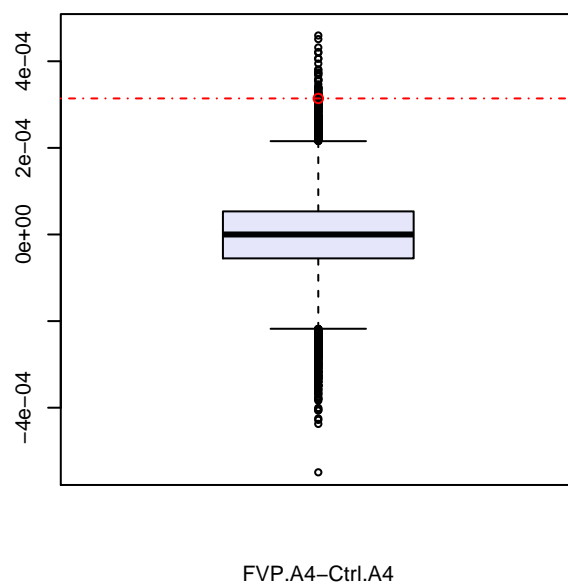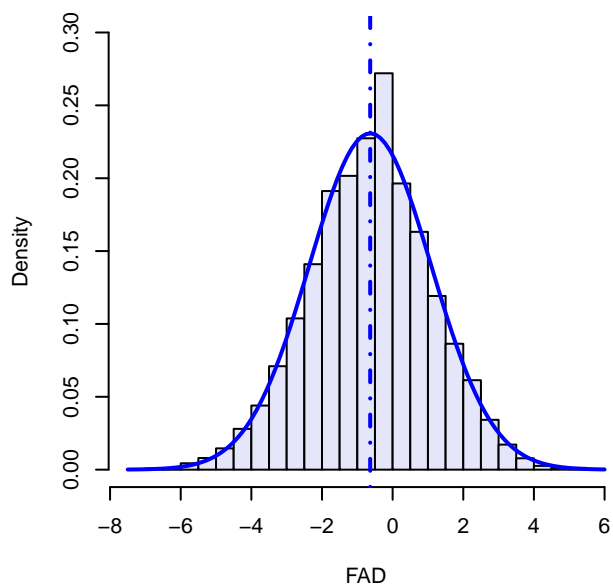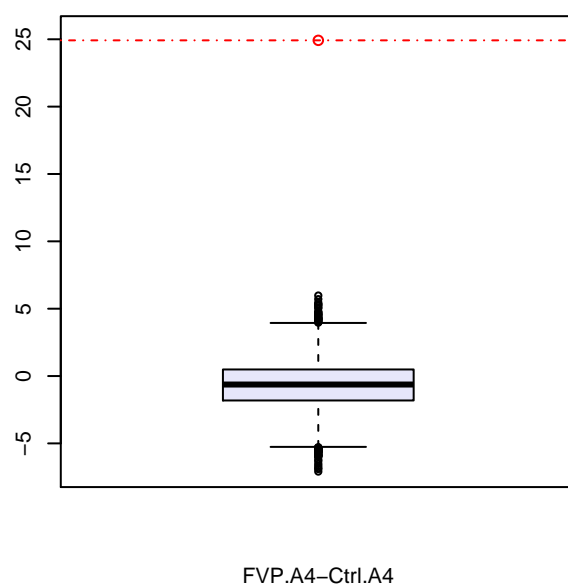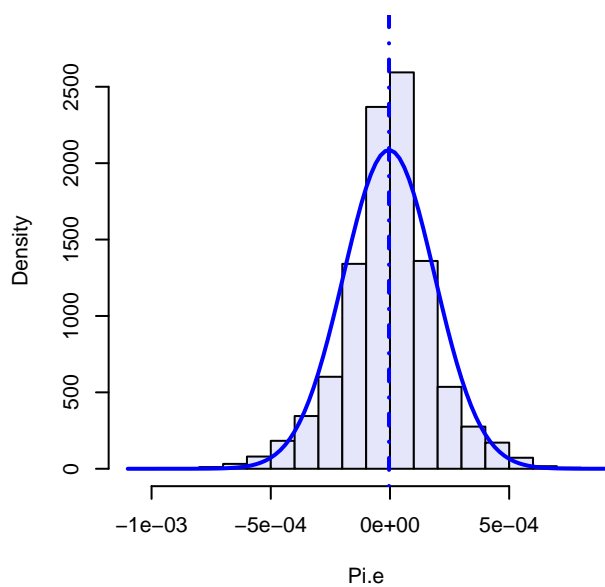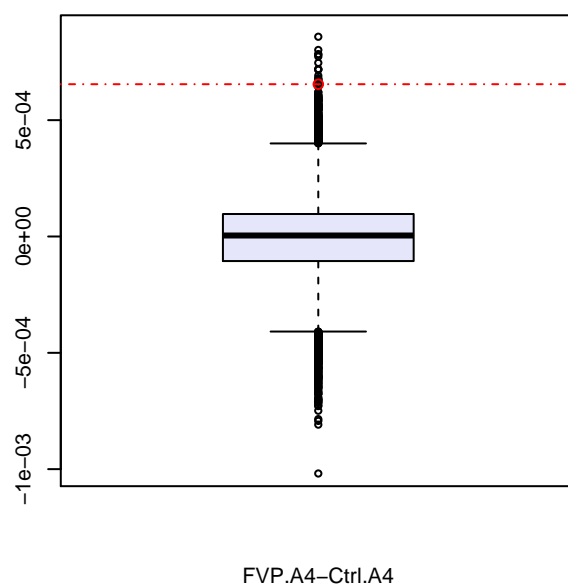

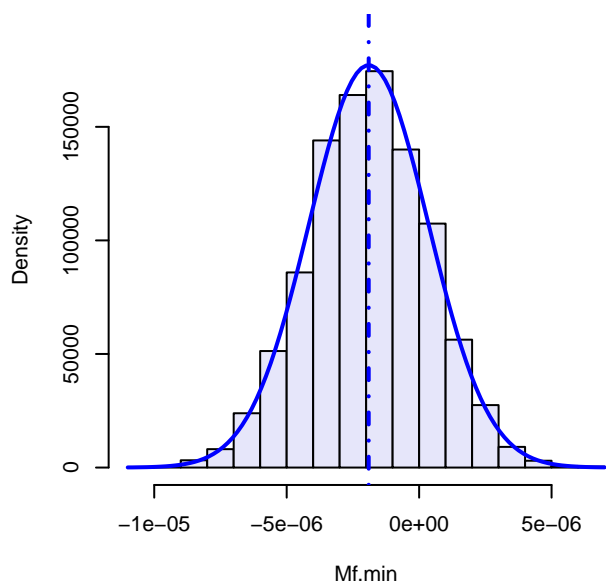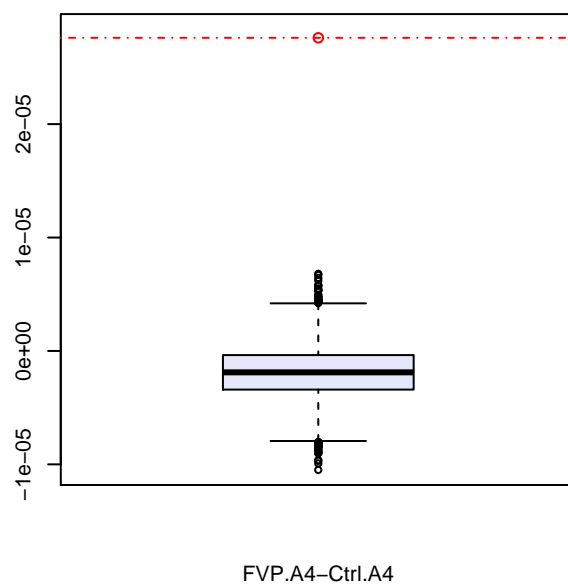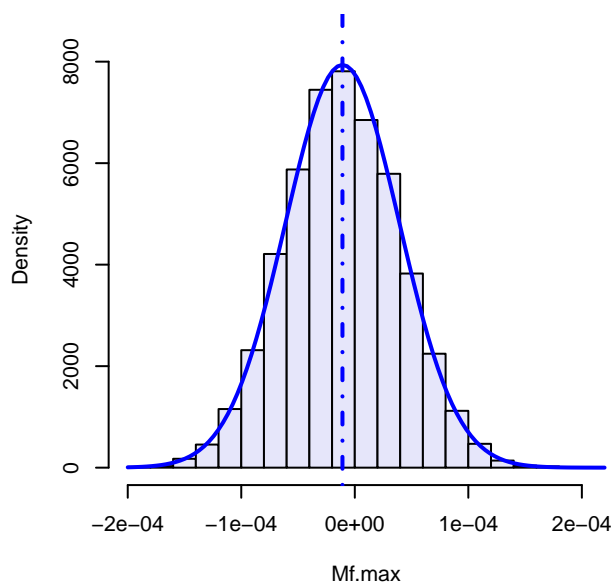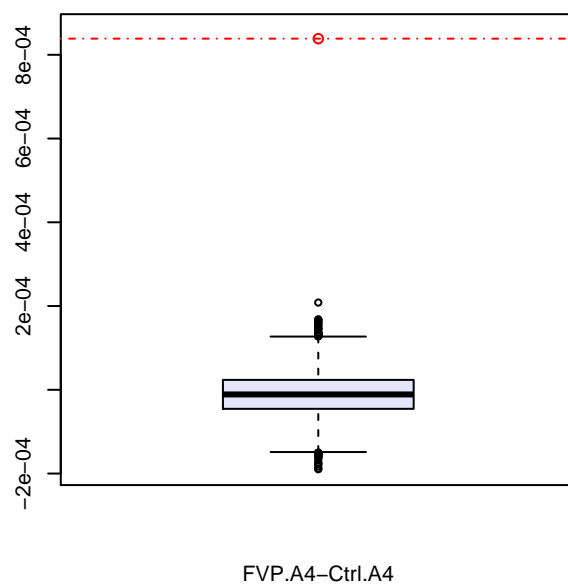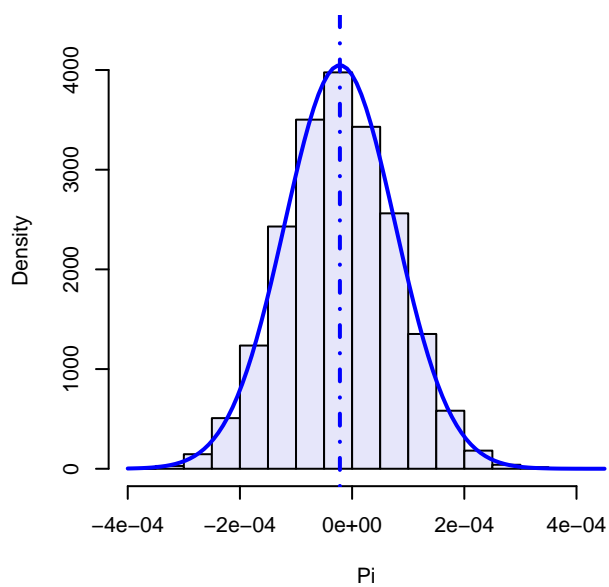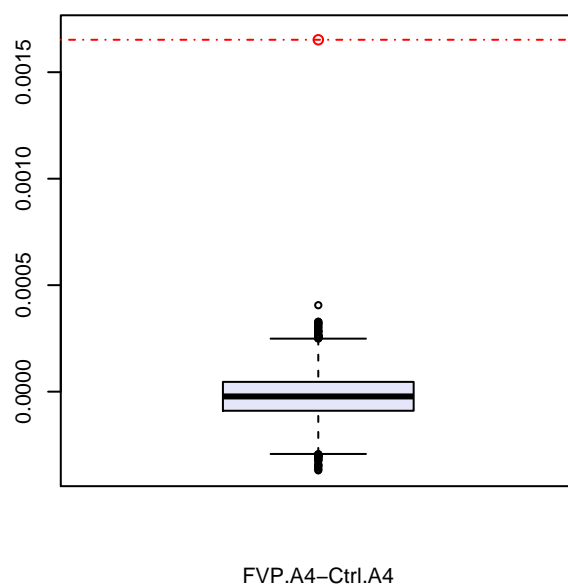

Supplement: S2 Fig — The diversity index is given in the abscissa, with the same abbreviations used in S1 Fig. Density means the probability density of the corresponding distribution. The panels on the right indicate the boxplot of null distribution bootstraped diversity differences, with observed difference as red dot and red dash-dot line. The distance from this line to the boxplot, in terms of boxplot width, is an illustration of the low p-values obtained. A1, A2 and A4 mean amplicons 1, 2 and 4, respectively. (PDF) [file pone.0164691.s002.pdf]
