# Supplementary material for: Lethal Mutagenesis of Hepatitis C Virus Induced by Favipiravir
Source: PLoS One. 2016 Oct 18;11(10):e0164691. doi: 10.1371/journal.pone.0164691 (PMC5068784; doi:10.1371/journal.pone.0164691)
Supplement: S1 Table — (PDF) [file pone.0164691.s004.pdf]

S1 Table. Oligonucleotides used for the ultra deep pyrosequencing analysis of HCV p0 subjected to three passages in the absence or presence of 400 µM favipiravir.

Primer Name	Sequence (5'-3')[a]	Sense	Position[b]	Tm (ºC)
**RT Oligonucleotide (5'-3')**				
**NS5A NakedT6R**	**ACTCAAAGGGTTGATTGGCAAC**	-	**7747-7726**	53
**Amplicon 1 Oligonucleotides (5'-3')**				
**13NS5A T1F**	GTTGTAAAACGACGGCCAGT**AAACCACGTCGCCCCTACTCAC**	+	**6130-6151**	58,6
**13NS5A T1R**	CACAGGAAACAGCTATGACC**AGGCGGACATTGCCAGAGAT**	-	**6474-6455**	53,8
**Amplicon 2 Oligonucleotides (5'-3')**				
**13NS5A T2F**	GTTGTAAAACGACGGCCAGT**TGACCACGCGCTGCCCTTGC**	+	**6426-6445**	60
**13NS5A T2R**	CACAGGAAACAGCTATGACC**AGACCTCATCCCGGAAAAACG**	-	**6788-6768**	54,4
**Amplicon 4 Oligonucleotides (5'-3')**				
**13NS5A T4F**	GTTGTAAAACGACGGCCAGT**ACATCACGGCGGAGACTGC**	+	**6891-6909**	55,4
**13NS5A T4R**	CACAGGAAACAGCTATGACC**AACGGTGGGCGGTTGGTAATC**	-	**7273-7253**	56,3
**MID 10 Oligonucleotide (5'-3')**				
**AFw10M13**	CGTATCGCCTCCCTCGCGCCA*TCAG*TCTCTATGCGGTTGTAAAACGACGGCCAGT	+	-	60
**BRv10M13**	CTATGCGCCTTGCCAGCCCGC*TCAG*TCTCTATGCGCACAGGAAACAGCTATGACC	-	-	60
**MID 11 Oligonucleotide (5'-3')**				
**AFw11M13**	CGTATCGCCTCCCTCGCGCCA*TCAG*TGATACGTCTGTTGTAAAACGACGGCCAGT	+	-	60
**BRv11M13**	CTATGCGCCTTGCCAGCCCGC*TCAG*TGATACGTCTCACAGGAAACAGCTATGACC	-	-	60

[a]Nucleotides in bold indicate the HCV-specific sequence; boxed nucleotides indicate universal M13 oligonucleotide. Nucleotides TCAG in italics indicate the sequencing key; these are the first four nucleotide to be sequenced and are located immediately downstream from the oligonucleotides A and B (supplier nomenclature, standard letters in the sequences); the underlined nucleotides indicate Roche's Validated Multiplex Identifiers (MID). MIDs 10 and 11 (in primers termed AFw and BRv in the first column) have been used to amplify HCV p0 passaged three times in the absence and presence of favipiravir, respectively.

[b]According to JFH-1 (accession number #AB047639)
