# Supplementary material for: Lethal Mutagenesis of Hepatitis C Virus Induced by Favipiravir
Source: PLoS One. 2016 Oct 18;11(10):e0164691. doi: 10.1371/journal.pone.0164691 (PMC5068784; doi:10.1371/journal.pone.0164691)
Supplement: S2 Table — (PDF) [file pone.0164691.s005.pdf]

S2 Table. Mutations, corresponding amino acid substitutions and point accepted mutation (PAM) of the NS5A-coding region in the mutant spectra HCV p0 subjected to three passages in the absence of drug analyzed by ultra deep pyrosequencing[a].

Amplicon A1			Amplicon A2			Amplicon A4		
**Mutation[b]**	**Amino acid substitution[c]**	**PAM 250**	**Mutation[b]**	**Amino acid Substitution[c]**	**PAM 250**	**Mutation[b]**	**Amino acid Substitution[c]**	**PAM 250**
A6260G[d]	**I259V[d]**	4	A6452G	**N62D**	2	G6959U	**V231L**	2
G6391A	**-**	-	A6491G	**T75A**	1	U7016C	**Y250H**	0
G6394A	**-**	-	A6578G	**T104A**	1	A7020G	**D251G**	1
A6452G	**N62D**	2	A6636G	**Q123R**	1	A7026G	**D253G**	1
A6453C	**N62T**	0	U6716C	**F150L**	2	A7074G	**E269G**	0
C6454A	**N62K**	1	C6753U	**A162V**	0	U7095C	**V276A**	0
			A6763U	**-**	-	G7117A	**M283I**	2
			G6766A	**-**	-	U7137C	**L290P**	-3
			C6767U	**P167S**	1	C7142U	**P292S**	1
			C6767G	**P167A**	1	A7148G	**I294V**	4
						U7149C	**I294T**	0
						A7150G	**I294M**	2
						C7152U	**P295L**	-3
						C7155U	**S296L**	-3
						A7158G	**E297G**	0
						G7159C	**E297D**	3
						U7160C	**C298R**	-4
						A7163G	**M299V**	2
						U7164C	**M299T**	-1
						U7167C	**L300P**	-3
						G7178A	**G304R**	-3
						A7207G	**-**	-
						A7215G	**D316G**	1
						G7240C	**-**	-
**Different mutations[e]**	**6**		**Different mutations[e]**	**10**		**Different mutations[e]**	**24**	
**Synonymous (%)[f]**	**2 (33.3)**		**Synonymous (%)[f]**	**2 (20)**		**Synonymous (%)[f]**	**2 (8.3)**	
**Non-synonymous (%)[f]**	**4 (66.6)**		**Non-synonymous (%)[f]**	**8 (80)**		**Non-synonymous (%)[f]**	**22 (91.6)**	

[a]The population analyzed correspond to passage 3 of the infections described in Fig. 1 and Table 1 of the main text.

[b]The HCV genome residue numbering corresponds to the Jc1-1 genome (accession number #AB047639).

[c]Amino acid residues (single letter code) are numbered from N- to the C-terminus of NS5A.

[d]Mutations (and deduced amino acid substitutions) at the NS4B-coding region.

[e]Number of different mutations found comparing the sequence of each individual clone with the consensus sequence.

[f]Number of different synonymous and non-synonymous mutations; their percentage is indicated in parenthesis.
