# Supplementary material for: Lethal Mutagenesis of Hepatitis C Virus Induced by Favipiravir
Source: PLoS One. 2016 Oct 18;11(10):e0164691. doi: 10.1371/journal.pone.0164691 (PMC5068784; doi:10.1371/journal.pone.0164691)
Supplement: S3 Table — (PDF) [file pone.0164691.s006.pdf]

S3 Table. Mutations, corresponding amino acid substitutions and point accepted mutation (PAM) of the NS5A-coding region in the mutant spectra HCV p0 subjected to three passages in the presence of favipiravir (T-705) 400 µM analyzed by ultra deep pyrosequencing[a].

**Amplicon A1**			**Amplicon A2**			**Amplicon A4**		
**Mutation[b]**	**Amino acid substitution[c]**	**PAM 250**	**Mutation[b]**	**Amino acid Substitution[c]**	**PAM 250**	**Mutation[b]**	**Amino acid Substitution[c]**	**PAM 250**
A6158G[d]	**T225A[d]**	1	A6452G	**N62D**	2	C6919U	**-**	-
G6160A[d]	**-**	-	C6469U	**-**	-	C6942U	**S225F**	-3
G6166A[d]	**-**	-	C6469A	**-**	-	U6943C	**-**	-
U6169C[d]	**-**	-	C6473A	**L69M**	4	C6952U	**-**	-
C6187U[d]	**-**	-	G6486A	**R73K**	3	C6955U	**-**	-
U6196C[d]	**-**	-	C6505U	**-**	-	G6959U	**V231L**	2
C6218U[d]	**-**	-	G6523A	**-**	-	G6974A	**A236T**	1
A6220G[d]	**-**	-	G6526A	**-**	-	G6989A	**A241T**	1
G6225A[d]	**R247K[d]**	3	A6527G	**T87A**	1	C6999U	**T244I**	0
A6226G[d]	**-**	-	C6529U	**-**	-	A7001G	**T245A**	1
A6242G[d]	**I253V[d]**	4	G6559A	**-**	-	C7003U	**-**	-
C6246U[d]	**T254I[d]**	0	C6562U	**-**	-	G7008A	**S247N**	1
G6250A[d]	**-**	-	G6565A	**-**	-	C7015U	**-**	-
A6260G[d]	**I259V[d]**	4	C6575U	**P103S**	1	U7016C	**Y250H**	0
C6268U[d]	**-**	-	C6577U	**-**	-	A7020G	**D251G**	1
C6298U	**-**	-	A6578G	**T104A**	1	A7026G	**D253G**	1
A6319G	**-**	-	A6582G	**N105S**	1	C7033U	**-**	-
C6335U	**-**	-	G6625A	**-**	-	A7049G	**M261V**	2
G6337A	**-**	-	A6632G	**T122A**	1	A7053C	**E262A**	0
A6338G	**T24A**	1	G6637A	**-**	-	G7064A	**A266T**	1
A6338U	**T24S**	1	G6646A	**-**	-	G7069A	**-**	-
C6340U	**-**	-	A6661G	**-**	-	A7074G	**E269G**	0
U6343C	**-**	-	C6665U	**-**	-	G7075A	**-**	-
C6373U	**-**	-	U6679C	**-**	-	C7077U	**P270L**	-3
C6379U	**-**	-	G6682A	**-**	-	G7081A	**-**	-
G6410A	**A48T**	1	U6692A	**C142S**	0	U7098C	**L277P**	-3

C6411U	**A48V**	0	C6694U	**-**	-	U7105C	**-**	-
A6452G	**N62D**	2	C6707U	**P147S**	1	G7111A	**-**	-
A6453C	**N62T**	0	G6739A	**-**	-	U7116C	**M283T**	-1
C6454A	**N62K**	1	C6742U	**-**	-	C7119U	**A284V**	0
			G6747A	**R160K**	3	G7124A	**E286K**	0
			C6753U	**A162V**	0	A7125G	**E286G**	0
			G6766A	**-**	-	G7129A	**-**	-
			C6767U	**P167S**	1	G7133A	**D289N**	2
						A7134G	**D289G**	1
						U7137C	**L290P**	-3
						G7141A	**-**	-
						C7142U	**P292S**	1
						C7144U	**-**	-
						U7145C	**S293P**	1
						A7148G	**I294V**	4
						U7149C	**I294T**	0
						A7150G	**I294M**	2
						C7151U	**P295S**	1
						C7152U	**P295L**	-3
						U7154C	**S296P**	1
						C7155U	**S296L**	-3
						A7158G	**E297G**	0
						G7159C	**E297D**	3
						U7160C	**C298R**	-4
						U7164C	**M299T**	-1
						G7165A	**M299I**	2
						C7166U	**L300F**	2
						C7169U	**P301S**	1
						A7172G	**R302G**	-3
						G7173A	**R302K**	3
						G7178A	**G304R**	-3
						G7179A	**G304E**	0

						U7183C	**-**	-
						A7215G	**D316G**	1
						U7217C	**Y317H**	0
						A7218G	**Y317C**	0
						G7225A	**-**	-
						G7240C	**-**	-
**Different mutations[e]**	**30**		**Different mutations[e]**	**34**		**Different mutations[e]**	**64**	
**Synonymous (%)[f]**	**18 (60)**		**Synonymous (%)[f]**	**21 (61.8)**		**Synonymous (%)[f]**	**18 (28.1)**	
**Non-synonymous (%)[f]**	**12 (40)**		**Non-synonymous (%)[f]**	**13 (38.2)**		**Non-synonymous (%)[f]**	**46 (71.9)**	

[a]The population analyzed correspond to passage 3 of the infections described in Fig. 1 and Table 1 of the main text.

[b]The HCV genome residue numbering corresponds to the JFH-1 genome (accession number #AB047639).

[c]Amino acid residues (single letter code) are numbered from N- to the C-terminus of NS5A.

[d]Mutations (and deduced amino acid substitutions) at the NS4B-coding region.

[e]Number of different mutations found comparing the sequence of each individual clone with the consensus sequence.

[f]Number of different synonymous and non-synonymous mutations; their percentage is indicated in parenthesis.
