# Supplementary material for: Lethal Mutagenesis of Hepatitis C Virus Induced by Favipiravir
Source: PLoS One. 2016 Oct 18;11(10):e0164691. doi: 10.1371/journal.pone.0164691 (PMC5068784; doi:10.1371/journal.pone.0164691)
Supplement: S4 Table — (PDF) [file pone.0164691.s007.pdf]

S4 Table. Mutations, corresponding amino acid and point accepted mutation (PAM) of the NS5B-coding region in the mutant spectra HCV p0 subjected to three passages in the absence or presence of 400 µM favipiravir (T-705)[a]

No drug			400 µM favipiravir		
**Mutation[b]**	**Amino acid substitution[c]**	**PAM 250**	**Mutation[b]**	**Amino acid substitution[c]**	**PAM 250**
C7703U	**P13S**	1	G 7690A	-	-
G7782A	**C39Y**	0	C 7705U	-	-
A7792U	-	-	G 7710A	**S15N**	1
U7967C	**Y101H**	0	G 7721A	**E19K**	0
A7983C	**K106T**	0	C 7735U	-	-
U8125C	-	-	G 7756A	-	-
U8279C	-	-	G 7843A	-	-
A8626G	-	-	U 7845C	**L60P**	-3
C8679U	**A338V**	0	G 7873A	-	-
U8716C	-	-	A 7875G	**D70G**	1
A8752G	-	-	C 7903U	-	-
G9118A	-	-	A 7945G	-	-
U9162C	**V499A**	0	C 7981U	-	-
G9163U	-	-	G 7987A	-	-
G9256A	-	-	G 8007A	**R114K**	3
U9305A	**L547I**	2	G 8008A	-	-
C9373U	-	-	U 8013C	**V116A**	0
			G 8108A	**D148N**	2
			G 8120U	**G152W**	-7
			C 8173U	-	-
			G 8186A	**A174T**	1
			G 8218A	-	-
			G 8222A	**V186I**	4
			C 8235U	**S190F**	-3
			A 8263C	**Q199H**	3
			C 8278U	-	-
			G 8405A	**E247K**	0
			C 8503U	-	-
			A 8560G	-	-
			C 8572U (x 2)	-	-
			C 8580U	**A305V**	0
			A 8588G	**I308V**	4

			A 8600G	**T312A**	1
			A 8633G	**I323V**	4
			C 8715A	**P350H**	0
			C 8723U	**P353S**	1
			C 8724U	**P353L**	-3
			C 8725U	-	-
			C 8767U	-	-
			C 8791U	-	-
			G 8808A (x 2)	**R381K**	3
			C 8812U	-	-
			C 8835U	**T390I**	0
			C 8854U	-	-
			U 8873A	**S403T**	1
			A 8887G	-	-
			C 8891U	-	-
			A 8918G	**T418A**	1
			C 8930U	**R422C**	-4
			C 8939U	-	-
			C 8971U	-	-
			G 8989A	-	-
			A 9013G	-	-
			A 9019G	-	-
			C 9022U	-	-
			C 9033U	**P456L**	-3
			A 9053G	**I463V**	4
			G 9070A	-	-
			U 9162C	**V499A**	0
			A 9307G	-	-
			G 9323A	**V553I**	4
			C 9331U	-	-
			A 9382G	-	-
			C 9397U	-	-
			C 9418U	-	-
			C 9419U	**L585F**	2
			G 9434A	**A590T**	1
			G 9439A	-	-
			G 9442A	-	-
**Different mutations[d]**	**17**		**Different mutations[d]**	**69**	
**Synonymous (%)[e]**	**10 (58.8)**		**Synonymous (%)[e]**	**38 (55.1)**	
**Non-synonymous (%)[e]**	**7 (41.2)**		**Non-synonymous(%)[e]**	**31 (44.9)**	

[a]The population analyzed correspond to passage 3 of the infections described in Fig. 1 and Table 2 of the main text.

[b]The HCV genome residue numbering corresponds to the JFH-1 genome (accession number #AB047639).

[c]Amino acid residues (single letter code) are numbered from N- to the C-terminus of NS5B.

[d]Data represent the total number of different mutations mutations found by comparing the sequence of each individual clone with the consensus sequence.

[e]Number of different synonymous and non-synonymous mutations; their percentage is indicated in parenthesis.
